# Identifying transcript 5′ capped ends in *Plasmodium falciparum*

**DOI:** 10.7717/peerj.11983

**Published:** 2021-08-25

**Authors:** Philip J. Shaw, Jittima Piriyapongsa, Pavita Kaewprommal, Chayaphat Wongsombat, Chadapohn Chaosrikul, Krirkwit Teeravajanadet, Manon Boonbangyang, Chairat Uthaipibull, Sumalee Kamchonwongpaisan, Sissades Tongsima

**Affiliations:** 1 National Center for Genetic Engineering and Biotechnology (BIOTEC), National Science and Technology Development Agency (NSTDA), Pathum Thani, Thailand; 2 National Biobank of Thailand (NBT), National Science and Technology Development Agency (NSTDA), Pathum Thani, Thailand

**Keywords:** 5′ capped nucleotide, Full-length cDNA, *Plasmodium falciparum*, Transcriptomics, Malaria, eIF4E

## Abstract

**Background:**

The genome of the human malaria parasite *Plasmodium falciparum* is poorly annotated, in particular, the 5′ capped ends of its mRNA transcripts. New approaches are needed to fully catalog *P. falciparum* transcripts for understanding gene function and regulation in this organism.

**Methods:**

We developed a transcriptomic method based on next-generation sequencing of complementary DNA (cDNA) enriched for full-length fragments using eIF4E, a 5′ cap-binding protein, and an unenriched control. DNA sequencing adapter was added after enrichment of full-length cDNA using two different ligation protocols. From the mapped sequence reads, enrichment scores were calculated for all transcribed nucleotides and used to calculate *P*-values of 5′ capped nucleotide enrichment. Sensitivity and accuracy were increased by combining *P*-values from replicate experiments. Data were obtained for *P. falciparum* ring, trophozoite and schizont stages of intra-erythrocytic development.

**Results:**

5′ capped nucleotide signals were mapped to 17,961 non-overlapping *P. falciparum* genomic intervals. Analysis of the dominant 5′ capped nucleotide in these genomic intervals revealed the presence of two groups with distinctive epigenetic features and sequence patterns. A total of 4,512 transcripts were annotated as 5′ capped based on the correspondence of 5′ end with 5′ capped nucleotide annotated from full-length cDNA data.

**Discussion:**

The presence of two groups of 5′ capped nucleotides suggests that alternative mechanisms may exist for producing 5′ capped transcript ends in *P. falciparum.* The 5′ capped transcripts that are antisense, outside of, or partially overlapping coding regions may be important regulators of gene function in *P. falciparum*.

## Introduction

Malaria remains the most widespread parasitic disease of humans, with over 200 million cases in 2018 (World Health Organization, 2019). The majority of severe malaria cases and deaths are attributed to *Plasmodium falciparum*, which is an obligate apicomplexan protist parasite of *Anopheles* mosquito and human hosts. Motile sporozoite stage parasites in the mosquito salivary gland enter the human host during blood feeding by the mosquito, which then migrate to the liver. Following a successful liver cell invasion, parasites multiply, develop and are then released into the bloodstream as merozoites capable of infecting mature erythrocytes. Infected erythrocytes are remodeled by the parasite as it develops through ring, trophozoite and schizont stages of the intraerythrocytic development cycle (IDC) before daughter parasites egress to invade new erythrocytes.

To facilitate discoveries of parasite molecular biology, such as finding new antimalarial drug and vaccine targets, the *P*. *falciparum* genome was sequenced in 2002 (Gardner et al., 2002). The finished reference genome comprises 5,280 protein-coding genes (Böhme et al., 2019). A total of 3,975 or more of the parasite’s genes are expressed in the IDC (Bozdech et al., 2003; Otto et al., 2010). However, the annotation of these genes is incomplete, in particular the associated RNA transcripts. Previous attempts to clone full-length complementary DNA (cDNA) corresponding to transcripts encountered problems with sequencing highly A/T rich flanking untranslated regions, which hampered efforts in mapping the transcript ends (Lu et al., 2007). Accurate mapping of transcript 5′ ends is challenging because reverse transcription synthesis of cDNA is initiated from the 3′ end of the RNA. Premature termination of cDNA synthesis can occur because of RNA degradation, inhibitory RNA secondary structure and limited processivity of the reverse-transcriptase enzyme (Das et al., 2001). Hence, the transcript 5′ end inferred from cDNA sequence can be an experimental artifact of incomplete cDNA. The 5′ ends of gene transcripts in *P*. *falciparum* are modified by the addition of a canonical eukaryotic 5′ cap m^7^G nucleotide (Ho & Shuman, 2001), which can be exploited for enriching 5′- capped mRNA (Shaw et al., 2007; Shaw et al., 2016).

One of the most widely used transcriptomic methods for enriching 5′-end fragments of eukaryotic mRNA is oligo capping, in which enzymatic treatments are used to append a synthetic oligonucleotide preferentially to mRNA 5′ ends that originally had a 5′ cap prior to reverse-transcription. The 5′ appended sequence serves as a primer for the synthesis of second strand cDNA so that the resulting cDNA is enriched for full-length fragments (Maruyama & Sugano, 1994). Full-length *P*. *falciparum* cDNA fragments were enriched by the oligo-capping method, cloned and sequenced in the Full-Malaria project (Watanabe et al., 2002). However, the limited coverage of *P*. *falciparum* genes prompted further study of transcript 5′ ends in *P*. *falciparum* using more comprehensive next-generation sequencing (NGS) approaches. Because NGS is limited to short read lengths, full-length cDNA fragments are generated by reverse-transcription proximal to the transcript 3′ end, typically by random priming. Throughout the rest of the manuscript, the term “full-length cDNA” refers to a cDNA fragment with sequence extending to the original transcript 5′ end, but not necessarily extending to the transcript 3′ end. Using an oligo-capping method adapted for NGS referred to as 5′ cap sequencing, full-length cDNAs covering the majority of *P*. *falciparum* genes were detected (Adjalley et al., 2016). However, despite the extensive sampling and depth of coverage employed, transcript 5′ ends were not detected for some genes known to be expressed in the IDC (Adjalley et al., 2016). One major reason for the missing 5′ end data could be 5′ end bias, in which some full-length cDNAs are inefficiently enriched. The oligo-capping enrichment method used in Adjalley et al. (2016) employs RNA ligase 1 enzyme, which is markedly less efficient for some RNA substrates (Fuchs et al., 2015).

Alternative strategies for enriching full-length cDNA are available that can complement oligo-capping and provide more comprehensive information of transcript 5′ ends. Switching mechanism at the 5′ end of the RNA transcript (SMART) is another widely used method for enriching full-length cDNA (Zhu et al., 2001). SMART is based on the propensity of reverse transcriptase enzymes to preferentially add extra cytosine nucleotides to the 3′ end of full-length first-strand cDNA. A template-switching oligonucleotide with complementary guanosines allows the reverse transcriptase to extend the cDNA using the oligonucleotide as a template. Second-strand cDNA synthesis is primed from the extended cDNA and the resulting double-stranded cDNA can be converted to a DNA library for NGS. Transcriptome-wide surveys of full-length *P*. *falciparum* cDNA enriched by SMART have been reported (Kensche et al., 2016; Chappell et al., 2020). Although a much greater proportion of the genome could be annotated as transcribed compared with previous transcriptomic studies, transcript 5′ ends were reported for 2,278 (Kensche et al., 2016) and 3,194 (Chappell et al., 2020) genes, suggesting that characterization of transcript 5′ ends in *P*. *falciparum* is not comprehensive by this approach. SMART enrichment employed in these studies studies is 5′ end-biased towards substrates with a terminal G (Wulf et al., 2019), which is reflected by the high frequency of G observed among 5′ end nucleotides (Chappell et al., 2020).

Cap-Trapper is an alternative to oligo-capping and SMART for enriching full-length cDNA in eukaryotes (Carninci et al., 1996). In this strategy, the diol group of the RNA 5′ cap is oxidized and coupled to biotin in the mRNA/cDNA products of reverse transcription. Incomplete cDNAs are depleted by ribonuclease digestion and the biotin-modified 5′ cap is used as a purification handle to enrich for full-length cDNA. In the Cap Analysis of Gene Expression (CAGE) method (Murata et al., 2014), NGS libraries are made from full-length cDNA fragments enriched by Cap-Trapper. To construct NGS library, adapters are ligated to the enriched first-strand cDNA. The 5′ adapter sequence used in CAGE is biased to enrich full-length cDNA with a “cap signature” guanosine derived from reverse-transcription of the 5′ cap m^7^G nucleotide (Ohtake et al., 2004). CAGE is technically demanding because the presence of the cap signature on full-length cDNA is strongly dependent on the reverse-transcription conditions (Wulf et al., 2019), and the efficiency of Cap-Trapper enrichment is strongly dependent on RNA purity and reaction conditions (Weiss & Curran, 2015). Although CAGE is the most accurate of current methods for mapping transcript 5′ ends in eukaryotes (Adiconis et al., 2018), it has not been widely used in non-metazoan organisms, including *P*. *falciparum*.

In this study, we developed a transcriptomic method for mapping of transcript 5′ capped ends. In our method, incomplete cDNAs are depleted enzymatically as in CAGE. Instead of chemical modification of 5′ capped ends, full-length cDNA is enriched using the eIF4E 5′ cap-binding protein, an enrichment strategy described as CAPture (Edery et al., 1995). We employ two different methods for adding NGS sequencing adapter to mitigate 5′ end bias. To increase signals of transcript 5′ ends, corresponding control transcriptomic libraries are prepared from cDNA samples before full-length enrichment. The degree of 5′ cap enrichment for each transcribed nucleotide is determined from the data using statistical models. To increase the power of detection, enrichment *P*-values are combined from replicate experiments (Promworn et al., 2017). Data were obtained with the new method from the ring, trophozoite and schizont stages of the *P*. *falciparum* IDC and used to annotate transcript 5′ ends. Published transcriptomic data from other studies were used together with the new data to annotate full-length 5′ capped transcripts.

## Materials & Methods

### Ethics statement

Human erythrocytes and serum were obtained from donors after providing informed written consent, following a protocol approved by the Ethics Committee, National Science and Technology Development Agency, Pathum Thani, Thailand, document no. 0021/2560.

### Parasite culture and mRNA enrichment

*Plasmodium falciparum* strain K1 (NCBI Taxonomy ID: 5839) was cultured *in vitro* in human O+ erythrocytes and medium containing pooled human serum and RPMI 1640 as described previously (Shaw et al., 2007). Cultured parasites were synchronized to ring stage by Percoll gradient enrichment of mature stages followed by sorbitol treatment (Lambros & Vanderberg, 1979). Parasites were harvested immediately (ring stage), 12 h (trophozoite stage) and 24 h (schizont stage) after sorbitol treatment. Parasites were liberated from the host cell by treatment with 0.1% (w/v) saponin. Parasite total RNA was obtained using Trizol reagent according to the manufacturer’s instructions (Invitrogen). Purified total RNA was stored in 75% ethanol at −80 °C before use. On the day of library preparation, total RNA was dried and then resuspended in nuclease-free water. Total RNA concentration was estimated by Nanodrop ND1000 measurement assuming A260 = 1.0 is equivalent to 40 µg/mL RNA. Up to 100 µg of total RNA was used for mRNA enrichment using a Dynabeads oligo dT25 mRNA kit (Thermo Scientific) or mRNA magnetic beads kit (New England Biolabs) followed by treatment with 1 unit of XRN-1 nuclease (New England Biolabs) for 30 min at 37 °C. XRN-1 enriched mRNA was purified by acidic phenol:chloroform extraction and ethanol precipitation. The mRNA was redissolved in 20 µL of nuclease-free water and genomic DNA was removed using a Turbo DNA-free kit following the manufacturer’s instructions (Ambion).

### Synthesis of cDNA for HiSeq libraries

*In vitro* synthesized spike-in RNA SIRV-Set 3 (Iso Mix E0/ERCC, Lexogen) was 5′ capped with Vaccinia capping system (New England Biolabs) following the manufacturer’s instructions. A 100–200 ng sample of parasite mRNA from ring or trophozoite stage (estimated by Nanodrop measurement) was mixed with 1.5 ng of 5′ capped SIRV-Set 3 RNA. Approximately the same amounts of parasite mRNA from schizont stages were used for reverse-transcription without the addition of spike-in RNA. The mRNA samples were incubated at 94 °C for 3 min in 1x first-strand cDNA synthesis buffer supplied with SuperscriptIV reverse transcriptase enzyme (Invitrogen) to fragment the RNA. Fragmented mRNA was chilled on ice for 5 min before reverse-transcription with 50 pmol of 5′ tagged random primer (RTNGS1 (Table S1) for adapter ligation method1, RTCIRC (Table S1) for adapter ligation method2; see below) with SuperscriptIV as described by the manufacturer in a final volume of 20 µL. After the addition of SuperscriptIV enzyme, reactions were incubated at 25 °C for 5 min, followed by heating to 37 °C for 30 min, 50 °C for 30 min, and then 70 °C for 15 min. First-strand cDNA was diluted to 100 µL with 10 mM Tris 1 mM EDTA and digested with 1 µL of RNaseA/T1 mix (Thermo Scientific) for 10 min at 37 °C to remove incompletely reverse-transcribed RNA. The mRNA/cDNA hybrid was purified using an equal volume of Agencourt^®^ AMPure^®^ XP beads (Beckman Coulter) and recovered from beads in 100 µL nuclease-free water. A 20 µL sample of purified mRNA/cDNA was kept for processing as the unenriched control sample.

### Synthesis of cDNA for MiSeq libraries

Samples of approximately 1 µg of mRNA (estimated by Nanodrop measurement) were incubated at 94 °C for 5 min in 1x first-strand cDNA synthesis buffer supplied with SuperscriptIII reverse transcriptase enzyme (Invitrogen) to fragment RNA. Fragmented mRNA was chilled on ice before reverse-transcription with 50 pmol of 5′ tagged random primer (RTNGS1 for method1, RTCIRC for method2) with SuperscriptIII as described by the manufacturer in a final volume of 20 µL. Method1 reverse-transcription reactions also contained 40 µM biotin-11-dUTP (Thermo Scientific) to label first-strand cDNA with biotin. After the addition of SuperscriptIII enzyme, reactions were incubated at 25 °C for 5 min, followed by heating to 37 °C for 30 min, and then 50 °C for 30 min. First-strand cDNA was diluted to 100 µL with 10 mM Tris 1 mM EDTA and digested with 1 µL of RNaseA/T1 mix (Thermo Scientific) for 10 min at 25 °C to remove incompletely reverse-transcribed RNA. The mRNA/cDNA hybrid was purified by proteinase K treatment, phenol:chloroform extraction and desalting on a Amicon-30 column (Ambion). The volume of desalted mRNA/cDNA hybrid was adjusted to 100 µL with nuclease-free water. A 20 µL sample of desalted mRNA/cDNA was kept for processing as the unenriched control sample

### Enrichment of full -length cDNA

*P*. *falciparum* eIF4E protein fused to glutathione-S transferase (GST-*Pf*eIF4E) (Shaw et al., 2007) was used for enriching full-length cDNA. GST-*Pf*eIF4E was expressed as recombinant protein in *Escherichia coli* and purified by SP-sepharose cation exchange chromatography as described previously (Shaw et al., 2007). Purified protein was buffer-exchanged using desalting columns and stored in a solution of 15% glycerol/RNase-free phosphate-buffered saline (PBS, Ambion). 5′ cap-binding activity of GST-*Pf*eIF4E protein was determined by m^7^GTP pulldown assay as described previously (Shaw et al., 2007), except that γ-aminophenyl-m^7^GTP (C10-spacer)-agarose (Jena Bioscience) was used as the affinity support. For each full-length cDNA enrichment, approximately 100 µg of SP-sepharose purified GST-*Pf*eIF4E protein was immobilized on 50 µL of glutathione magnetic beads (Thermo Scientific) in PBS. The glutathione magnetic beads were washed four times with 0.5 mL of PBS to remove unbound protein and then resuspended in 100 µL of PBS. The remainder of the purified mRNA/cDNA sample was incubated with bead-bound GST-*Pf*eIF4E protein for 20 min at 25 °C with agitation (750 rpm Thermomixer). The beads were then washed thrice with 0.5 mL PBS and the mRNA/cDNA eluted in 100 µL of 1% sodium dodecyl sulfate/0.2 M sodium chloride. The eluted mRNA/cDNA was purified with an equal volume of Agencourt^®^ AMPure^®^ XP beads (Beckman Coulter) and recovered from beads in 20 µL of nuclease-free water. RNA was removed from enriched and unenriched cDNA samples by alkaline hydrolysis (15 min treatment at 65 °C with 0.2 N NaOH). Alkaline-treated cDNA was neutralized with an equal volume of 1 M HEPES pH 7.4 solution. First-strand cDNA was purified with an equal volume of Agencourt^®^ AMPure^®^ XP beads (Beckman Coulter) and recovered from beads in 20 µL of nuclease-free water.

### DNA adapter ligation to cDNA

In some experiments, sequencing adapter was added by cDNA tailing followed by double-stranded adapter ligation (hereafter referred to as method1). A ribo-G tail was added to the purified single-stranded cDNA primed with RTNGS1 (Table S1) using terminal transferase (TdT, New England Biolabs). The tail length is limited to four Gs (Schmidt & Mueller, 1996). The TdT tailing reactions contained 2 mM GTP, 0.25 mM CoCl_2_, 1 unit of TdT enzyme in 1x TdT enzyme buffer and first-strand cDNA. TdT reactions were performed in 20 µL reaction volumes for 20 min at 37 °C. Ribo-tailed cDNA was purified with an equal volume of Agencourt^®^ AMPure^®^ XP beads (Beckman Coulter) and recovered from beads in 20 µL of nuclease-free water.

Double-stranded DNA adapter was made by combining 4 nmol each of NGS1 and NGS1COMP oligonucleotides (Table S1) in a volume of 100 µL with 10 mM NaCl and 10 mM Tris pH 8.0. The NGS1 oligonucleotide is 5′-phosphorylated to act as a donor in ligation and blocked with a three-carbon spacer at the 3′ end to prevent concatemerization of adapters. Annealing was accomplished by heating the mixture for 3 min at 80 °C followed by slow cooling to 25 °C (−0.1 °C/min). Double-stranded DNA adapter was ligated to first-strand cDNA using T4 RNA ligase 2 (RNL2, New England Biolabs). The ligation reactions contained 40 pmol of DNA adapter, 7.5% (w/v) PEG_6000_, 1x RNL2 enzyme buffer, 2.5 units of RNL2 enzyme and first-strand cDNA in a volume of 20 µL. The ligation reactions were performed for 99 cycles of 37 °C for 30 s, 22 °C for 30 s. After the completion of the RNL2 adapter ligation reactions, cDNA for HiSeq sequencing was diluted to 100 µL with nuclease-free water and purified with an equal volume of Agencourt^®^ AMPure^®^ XP beads (Beckman Coulter). Purified cDNA was recovered in 50 µL of nuclease-free water. For adapter-ligated cDNA samples sequenced on the MiSeq platform, ligation products were purified using 25 µL Streptavidin M280 magnetic beads following the manufacturer’s recommendations (Invitrogen). Synthesis of second-strand cDNA was performed on the beads using T7 DNA polymerase following the manufacturer’s recommendations (New England Biolabs). The beads were resuspended in 50 µL of T7 reaction mix (0.3 mM dNTPs, 2.5 units T7 DNA polymerase, 1x T7 DNA polymerase buffer). The reaction was performed for 15 min at 37 °C. The reaction was terminated by washing the beads in 0.4 mL of washing solution (40 mM Tris pH 8.0, 10 mM MgCl_2_). The second-strand cDNA was eluted from the beads by heating for 3 min at 99 °C in a suspension of 50 µL 1x Sodium Saline Citrate (150 mM sodium chloride, 15 mM trisodium citrate pH 7.0).

An alternative adapter ligation strategy (hereafter referred to as method2) was also employed. Purified first-strand cDNA primed with RTCIRC in which RNA had been removed by alkaline treatment was circularized with 100 U of CircLigaseII enzyme, 2.5 mM MnCl_2_, 1 M betaine, and 1x CircLigaseII buffer in a reaction volume of 20 µL as recommended by the manufacturer (Epicentre). The reaction was incubated at 60 °C for 1 h and the reaction terminated by heating to 80 °C for 10 min. The reaction was diluted to 100 µL with nuclease-free water and cDNA purified using half the volume of Agencourt^®^ AMPure^®^ XP beads (Beckman Coulter). Purified cDNA was recovered in 50 µL of nuclease-free water.

### Library purification and Illumina sequencing

Adapter-ligated cDNA was used as a template for PCR amplification to make sequencing library. For samples sequenced using the HiSeq platform, PCRs contained adapter-ligated cDNA template (1 µL), 2.5 pmol each of PE1 and ScriptSeq primers (Table S1), 200 µM dNTPs, 0.625 U PrimeSTAR^®^ GXL DNA polymerase (Takara) and 1x buffer supplied with the enzyme in a reaction volume of 25 µL. The PCR program used was 98 °C for 30 s, followed by 18–25 cycles of 98 °C for 10 s, 68 °C for 60 s and a final extension of 68 °C for 5 min. For samples sequenced using the MiSeq platform, PCRs contained adapter-ligated cDNA template (1 µL), 12.5 pmol each of PE1 and ScriptSeq primers, 200 µM dNTPs, 2 mM MgCl_2_, 0.5 units of Platinum *Taq* enzyme (Invitrogen) and 1x Platinum *Taq* buffer in a reaction volume of 50 µL. The PCR program used was 94 °C for 90 s, followed by 18–25 cycles of 94 °C for 10 s, 60 ° C for 60 s. The optimal number of PCR cycles for each sample was determined empirically by visualization of products separated by 1.5% agarose gel electrophoresis from pilot reactions conducted with varying numbers of cycles. Amplified DNA fragments 400–600 bp in size were excised from the gel and purified using a MinElute gel extraction kit (Qiagen), with the modification that agarose was solubilized in QG buffer at room temperature to prevent DNA denaturation. DNA was quantified using a Qubit™ dsDNA HS Assay kit (Invitrogen). Libraries were pooled in equimolar ratios according to the ScriptSeq index primer recommendations (Illumina).

Pooled libraries were submitted to Novogene AIT (Singapore) for Illumina standard protocol sequencing on one lane of a HiSeq2000 flow-cell (Illumina), or to the Faculty of Medicine, Chulalongkorn University, Bangkok, Thailand for MiSeq sequencing. For MiSeq sequencing, pooled libraries were processed with 1% Illumina phiX174 control and loaded onto MiSeq v3 flow cells at 10 pM. 150 bp paired-end sequencing was performed using the standard sequencing primers as recommended by the manufacturer (Illumina). For MiSeq sequencing of libraries prepared using adapter ligation method 1, the sequencing “recipe” for the MiSeq instrument was modified to perform the first four sequencing cycles as “dark”, meaning no data are recorded for the purpose of identifying sequencing clusters. This modification was necessary since the homopolymer tail added by TdT during library construction provides insufficient diversity for generating a high density of clusters. Without this modification, there will be data loss in the cluster identification step (clusters passing filter quality control) of Illumina sequencing.

### Analysis of transcriptomic data from 5′ capped nucleotide enrichment experiments

The 5′ end of read1 from cDNA adapter ligation method1 data obtained using the HiSeq platform was trimmed of homopolymer sequence added by TdT using Cutadapt 1.18 (Martin, 2011). Read trimming was not performed for method 1 data obtained using the MiSeq platform, as signals for bases 1–4 were not recorded for this platform. Adapter sequence in the 3′ end of reads was trimmed and low-quality reads were removed using fastp with default settings (Chen et al., 2018). Preprocessed paired read data were aligned to the combined *P*. *falciparum* 3D7 v3.2 (Böhme et al., 2019) / SIRVomeERCCome (Lexogen) reference genome using HISAT2 (Kim et al., 2019), in the spliced mode guided by the *P*. *falciparum* / SIRVomeERCCome genome annotation with maximum intron length limited to 5,000 bp and other settings as default. Alignment summary statistics are shown in Table S2. To assess biological reproducibility, gene expression was determined for annotated *P*. *falciparum* genes using unenriched .bam files from each experiment as input to StringTie (Pertea et al., 2016). Gene expression values reported as transcripts per million were log_10_ transformed and used for sample pairwise Pearson correlations using the ggcorrplot package in R (Kassambra, 2019).

For analysis of 5′ cap nucleotide enrichment, .bam alignment files were used as input for the ToNER program (Promworn et al., 2017) with default settings, *i.e.,* depth value calculated from the read start position, pseudo-read added to depth values at all transcribed nucleotides and no filtering of positions for statistical modeling. This program calculates enrichment scores from paired feature-enriched and unenriched transcriptomic data. The enrichment scores are then transformed and fitted to a normal distribution by the Box–Cox procedure, and statistics of enrichment for each nucleotide reported. To increase statistical power, combined *P*-values from independent enrichment experiments were calculated by Fisher’s method. The enrichment signals at individual nucleotides were clustered into genomic intervals representing the 5′ capped ends of major transcripts using the CAGEr package (Haberle et al., 2015) as follows. Clustering of nucleotide signals in CAGEr requires integer counts, which are referred to in the CAGEr manual as tags. To generate tags for clustering, the reciprocal of enrichment *P*-value was rounded to the nearest integer. To militate against program abort owing to the constraint of maximum allowed integers in R, the maximum tag count was set as 1e7. Tags were clustered with the distclus function in CAGEr with no normalization and maximum distance between merged clusters of 20 nt. The dominant_ctss position in each cluster reported by CAGEr was annotated as a 5′ capped nucleotide. CAGEr clusters overlapping known 5′ ends in SIRV spike-in RNAs (assigned as true positives) were identified with BEDTools (Quinlan & Hall, 2010). The sum of tags (tpm) in each CAGEr cluster mapping to SIRVomeERCCome reference was used for Receiver Operator Characteristic (ROC) analysis with the plotROC package (Sachs, 2017). The optimal tpm value for filtering CAGEr clusters was identified using the OptimalCutpoints R package (López-Ratón et al., 2014) with default settings. For quantitative analysis of External RNA Controls Consortium (ERCC) RNA spike-ins, the CAGEr clusters reported from tag input of combined *P*-values of enrichment from four experiments were used. An RNA 5′ end was called as detected if a CAGEr cluster overlapped its known location. The empirical cumulative distribution functions of detected and undetected 5′ ends with respect to natural log of ERCC RNA concentration reported by the manufacturer (Lexogen) were determined using the base R ecdf function. The distributions of detected and undetected 5′ end groups were compared by two-sample Kolmogorov–Smirnov test in R. The natural log tpm values for CAGEr clusters overlapping detected 5′ ends were plotted against the natural log concentration of each RNA using the ggplot2 package (Wickham, 2009) in R.

### Analysis of genomic context in the vicinity of 5′ capped nucleotides

5′ capped nucleotide signals detected as CAGEr clusters above threshold (tpm >32) in separate stages of *P*. *falciparum* development (including cluster signals within 100 bp of each other) were merged into 17,961 non-overlapping genomic intervals using BEDTools (Quinlan & Hall, 2010). Within each interval, the dominant_ctss nucleotide with highest integer count (tpm.dominant_ctss reported by CAGEr) across the sampled developmental stages was annotated as the representative 5′ capped nucleotide for genomic analysis. Genomic sequences flanking 100 bp on either side of the 17,961 5′ capped nucleotide (test set) and 17,961 random positions selected using BEDTools (Quinlan & Hall, 2010) (control set) were obtained from the *P*. *falciparum* 3D7 v3.2 reference genome (Böhme et al., 2019) using the seqPattern package in R (Haberle, 2020). Sequence analysis was performed using the kpLogo program (Wu & Bartel, 2017) using the control set sequences as background and other settings as default. For analysis of epigenetic features, publicly available datasets from published studies were processed to construct genome coverage files (bigwig format) as described below in sub-section analysis of external epigenetic data. Plots of average coverage for each feature were made using the genomation package (Akalin et al., 2015) in R. To mitigate the effect of extreme values, the top and bottom 5% of scores were clipped using the winsorize function in the genomation package.

### Mismatched base analysis of -1 positions

The first base of all uniquely aligned reads (with secondary alignments removed from alignment .bam files using SAMtools) was extracted from all datasets. For reads mapping to the reference (–) strand, the complement of the first base was taken. Bases mismatched to the reference sequence (corresponding to soft-clipped bases in the alignment files) were counted for two groups. The first group comprised reference positions one base upstream of dominant 5′ capped nucleotides in non-overlapping genomic intervals (17,961 positions), and the second group all other reference positions. Contingency tables were constructed for each dataset for the count of the tested mismatched bases and all other mismatched bases for both groups of nucleotides (Table S3). One**-**tailed Fisher**’**s exact tests were performed of the alternative hypothesis that the true odds ratio is greater than 1. One**-**tailed tests were performed because mismatched bases under**-**represented in the first position of reads are not of interest. Bonferroni**-**corrected *P***-**values less than 0**.**001 were considered significant. For analysis of reference sequence patterns at -1 positions with high counts of mismatched reads, positions with 10 or more aligned reads and >50% of reads with mismatched first base in any dataset were selected.

### Unsupervised cluster analysis of 5′ capped nucleotides

17,961 5′ capped nucleotides in non-overlapping genomic intervals annotated from our data (see above) were clustered using epigenetic data, including occupancies of H2A.Z, H3K9 acetylation and H3K4 trimethylation (Bártfai et al., 2010) and H2B.Z (Hoeijmakers et al., 2013). For details of how chromatin mark occupancies were determined, see below in sub-section analysis of external epigenetic data. Principal Component Analysis (PCA) scores were calculated using the MacroPCA package in R (Hubert, Rousseeuw & VandenBossche, 2019). The data were pre-processed by transformation with natural logarithms, scaling by unit variance, means-centering and removal of rows with too many missing values. The first three principal components explained 83.3% of the variance; hence, the PCA scores from the first three principal components were used for clustering. To determine if more than one cluster existed in the data, statistical tests of unimodality were performed on PC1 scores using the multimode package in R (Ameijeiras-Alonso, Crujeiras & Rodríguez-Casal, 2019). Test *P*-values less than 0.05 were considered significant. Clustering was performed using the cross-entropy clustering (CEC) package in R (Tabor & Spurek, 2014; Spurek et al., 2017). The algorithm employed in this program is a hybrid of k-means and Gaussian mixed model, and can thus separate clusters with a variety of shapes. The settings used for CEC clustering were: Gaussian distribution models unconstrained, nstart =1000, initial clusters =5 and card.min =20%. To determine independently how many relevant clusters are present, the PCA scores from PC1, PC2 and PC3 were analysed using the NbClust package in R (Charrad et al., 2014). Cluster indices were determined using the Euclidean distance matrix and k-means method for *n* = 2 to *n* = 6 clusters. All indices were calculated except Gplus and Tau, which could not be determined owing to computational constraint, and Gamma which is only applicable for hierarchical clustering.

### Analysis of external transcriptomic data

To assess the reproducibility of 5′ capped nucleotides in *P*. *falciparum*, data were analyzed from independent published studies using different methods for 5′ capped nucleotide enrichment. *P*. *falciparum* 5′ cap sequencing data reported in Adjalley et al. (2016) were downloaded from the NCBI GEO database series under accession number GSE68982 using sratoolkit.2.10.0-ubuntu64 (SRA Toolkit Development Team, 2014). The read1 fastq files were pre-processed with UMI-tools (Smith, Heger & Sudbery, 2017) to move the 8 bp unique molecular index to the read header. Pre-processed paired data files were filtered and trimmed of 3′ adapter sequence using fastp (Chen et al., 2018) with default settings. Data from *P*. *falciparum* SMART-enrichment experiments reported in Kensche et al. (2016), European Nucleotide Archive accession numbers ERR861692 and ERR861693, were uploaded to the public server at usegalaxy.org. Read1 were filtered and trimmed using the Barcode Splitter tool to retain reads that started with smartseq2 adapter (AAGCAGTGGTATCAACGCAGAGTACATGGG), allowing up to three mismatches or indels. The filtered read1 and read2 from 5′ cap sequencing and SMART-enrichment experiments were processed using fastp (Chen et al., 2018) with default settings. Processed paired 5′ cap sequencing and SMART-enrichment data from fastp were aligned to the *P*. *falciparum* 3D7 v3.2 reference genome (Böhme et al., 2019) using HISAT2 (Kim et al., 2019) in the spliced mode guided by the genome annotation (PlasmoDB release 44) with maximum intron length limited to 5,000 bp and other settings as default. PCR duplicates in 5′ cap sequencing data were removed using UMI-tools (Smith, Heger & Sudbery, 2017) with the–unmapped-reads =use option. Unmapped and read2 reads were removed from .bam files and a merged .bam file was created from all experiments in each study using SAMtools (Li et al., 2009).

The filtered .bam files from individual experiments were used to obtain read depth at each position in the genome with BEDTools (Quinlan & Hall, 2010), with -strand and -5 options. The read depth values were used as ctss input for CAGEr (Haberle et al., 2015) for detection of 5′ capped nucleotide signals. CAGEr was run with the distclus function, no normalization and maximum distance between merged clusters of 20 nt. The CAGEr analysis results of 5UTR-seq experiments in *P*. *falciparum* were obtained from Table S9 reported in a previous study (Chappell et al., 2020). A merged .bam file of aligned data from all 5UTR-seq experiments was provided by Dr. Lia Chappell.

The dominant_ctss nucleotide genomic locations and the corresponding tpm.dominant _ctss values reported for CAGEr clusters in each dataset were concatenated and used to construct .bed and .bedgraph files of 5′ capped nucleotides for each study. In addition to CAGEr clusters, the read depth at each position in the genome was obtained from the merged .bam file from all experiments in each study using BEDTools (Quinlan & Hall, 2010) with -strand and -5 options. To assess the agreement of 5′ cap nucleotide assignment in our data with other studies, agreement was scored if the depth value from the combined .bam file of all experiments in the same study was two or greater.

Direct RNA sequencing data from *P*. *falciparum* obtained using the Oxford Nanopore MinION platform reported in Lee et al. (2021) under SRA accession number SRR11094274 were uploaded to the public server at usegalaxy.org and aligned to the *P*. *falciparum* 3D7 v3.2 reference genome (Böhme et al., 2019) using Minimap2 (Li, 2018) with settings long-read spliced alignment, maximum intron 5 kb, and search GT-AG on the transcript strand only. The .bam alignment file was used as input to the Full-Length Alternative Isoform analysis of RNA (FLAIR) program (Tang et al., 2020a) with the–nvrna option using reference genome sequence and annotation (PlasmoDB release 44) for correction of isoform sequences. The corrected and inconsistent isoform sequences outputted by FLAIR were concatenated to create a single transcript annotation .gtf file. To assess whether transcripts annotated by FLAIR were 5′ capped, the transcript 5′ end locations were compared with 5′capped nucleotides annotated from 5′ cap enrichment data using BEDTools (Quinlan & Hall, 2010). Genomic locations in .bed file format were constructed for each 5′ cap enrichment study from the dominant_ctss nucleotide reported in all CAGEr clusters. FLAIR transcripts were considered full-length (5′ capped) if the transcript 5′ end was 20 nt or closer to a dominant_ctss nucleotide.

### Analysis of external epigenetic data

Epigenetic data for analysis of 5′ capped nucleotide genomic context were obtained from published studies, including chromatin immunoprecipitation sequencing (ChIP-seq) data reported in Bártfai et al. (2010), Hoeijmakers et al. (2013), Karmodiya et al. (2015), Lu et al. (2017), Tang et al. (2020b), Bhowmick et al. (2020) and micrococcal nuclease-digested chromatin sequencing (MNase-seq) reported in Kensche et al. (2016) were obtained from the NCBI database under series accession numbers GSE23867, GSE39702, GSE63369, GSE85478, PRJNA612099, GSE142803 and GSE66185, respectively. The .csfasta files in accession GSE63369 were converted to .fastq using Cutadapt 1.18 (Martin, 2011) and aligned to the *P*. *falciparum* 3D7 v3.2 reference genome (Böhme et al., 2019) using bowtie−1.2.3-linux-x86_64 (Langmead et al., 2009) with the following options: -C -v 2 –best –strata m 3. The data from the other studies were uploaded to the public server at usegalaxy.org and processed using fastp (Chen et al., 2018) with default settings. For MNase-seq data, read length was trimmed with fastp to a maximum of 72 bp. Processed read data were aligned to the *P*. *falciparum* 3D7 v3.2 reference genome (Böhme et al., 2019) using bowtie2 (Langmead & Salzberg, 2012) with default settings. The .bam files for experimental replicates or multiple runs of the same library were merged using SAMtools (Li et al., 2009).

In order to calculate nucleosome occupancy from MNase-seq data at each sampled timepoint of development, sonicated genomic DNA control datasets with matching insert size distributions were created. The count of fragments with the same insert size in the alignment file generated from each MNase-seq experiment was obtained using SAMtools (Li et al., 2009). Next, insert size distributions were determined from the count of inserts in 31 bins of varying insert size (32–52, 53–72, 73–82, 83–92, 93–102, 103–12, 113–122, 123–132, 133–142, 143–152, 153–162, 163–172, 173–182, 183–192, 193–202, 203–212, 213–222, 223–232, 233–252, 253–272, 273–292, 293–312, 313–332, 333–352, 353–372, 373–392, 393–412, 413–432, 433–452, 453–472, and 473–500 bp). Alignment .bam files of sonicated genomic DNA control with defined insert sizes were made using BamTools (Barnett et al., 2011). The alignment files of sonicated genomic DNA control with defined insert sizes were randomly sampled to the equivalent depth in the MNase-seq experimental dataset and merged to create a genomic DNA control with matching insert size distribution using SAMtools (Li et al., 2009).

Normalization factors for each sample dataset from all epigenetic experiments were calculated by dividing the reference genome size (23,332,839 bp) by the total depth of coverage in .bam files obtained using SAMtools (Li et al., 2009). The read depth at each position in the genome was obtained using BEDTools (Quinlan & Hall, 2010). Nucleotide occupancy for each epigenetic feature of interest was calculated as the ratio of normalized read depth in each test dataset to developmental stage-matched control. To prevent division by zero, a pseudo depth value equal to 0.001 was added to every position for test and control datasets. For ChIP-seq data reported in Lu et al. (2017), RNA polymerase II (RNApolII) occupancy was calculated by subtraction of timepoint-matched normalized IgG negative control read depth from normalized ChIP-seq read depth. Nucleotide occupancy values were used to create genome coverage files in .bigwig format using the bedGraphToBigWig tool (Kent et al., 2010).

Genomic intervals annotated as *P*. *falciparum* accessible chromatin from Assay for Transposase-Accessible Chromatin (ATAC-seq) experiments were obtained from the supplementary information files reported in Toenhake et al. (2018), Ruiz et al. (2018) and Yin et al. (2020). The accessible chromatin intervals reported in each study were intersected using BEDTools (Quinlan & Hall, 2010).

### Data availability

Custom scripts written for analysis of transcriptomic data are available from the GitHub repository: https://github.com/BSI3/5CAPture-seq. Sequencing data generated in this study are available from the National Center for Biotechnology Information (NCBI) Gene Expression Omnibus (GEO) database under series accession number GSE103036.

## Results

### Outline of the method for identifying 5′ capped nucleotides

**Figure 1:**
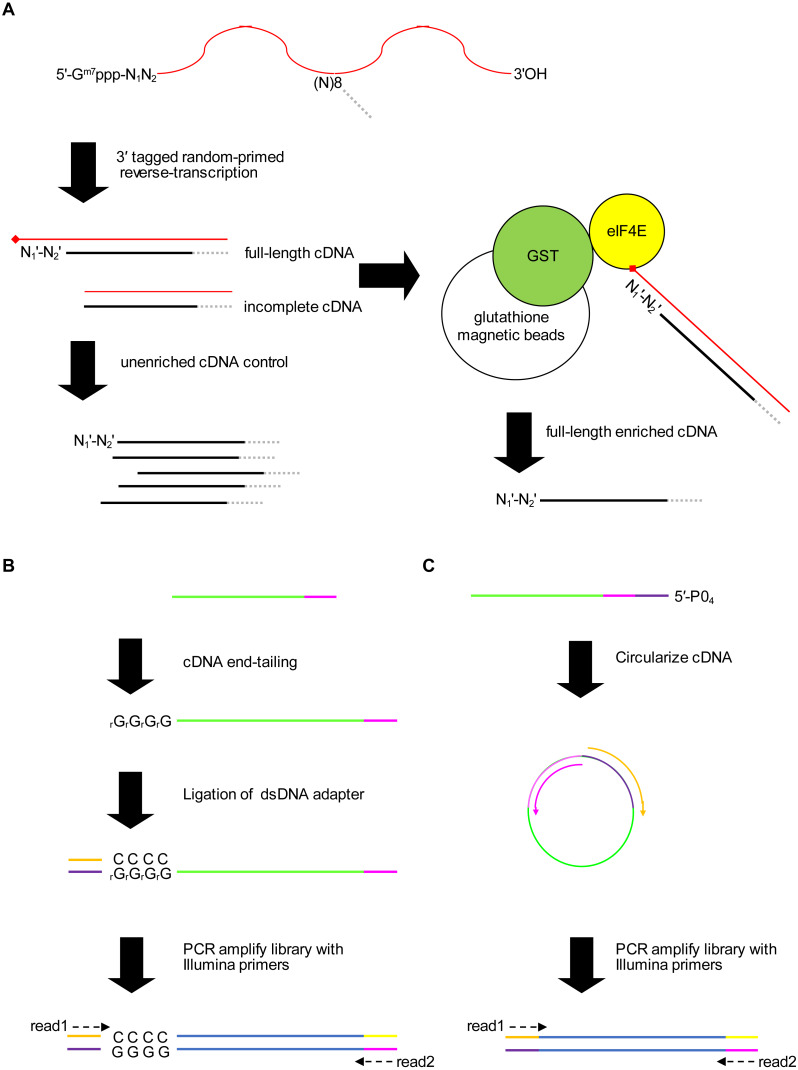
Schematic of the 5′ cap enrichment transcriptomic method. (A) 5′ capped mRNA is reverse-transcribed using a random primer with 3′ sequence tag (magenta). Full-length cDNA extending to the 5′ cap structure (lollipop) terminates in a 3′ nucleotide (N${}_{1}^{{^{\prime}}}$) complementary to authentic 5′ mRNA end nucleotide (N_1_). A portion of the cDNA sample is reserved as unenriched for generating a control library, whereas the remainder is enriched for full-length cDNA by CAPture (Edery et al., 1995). Recombinant protein of glutathione S-transferase (GST) fused to 5′ cap binding protein (eIF4E) is immobilized on glutathione magnetic beads. Incomplete cDNA is removed by washing and full-length cDNA eluted from the beads. Unenriched control and full-length enriched cDNA are processed in parallel for 5′ adapter ligation and construction of sequencing libraries. (B) 5′ adapter ligation method1. First-strand cDNA primed using RTNGS1 (Table S1) is 3′ tailed using terminal transferase and rGTP. Up to four riboguanosines (rG) are added to the 3′ end of the cDNA (Schmidt & Mueller, 1996). Double-stranded DNA adapter with overhanging complementary Cs is ligated to the first-strand cDNA using RNA ligase 2 enzyme. The adapter is made by annealing top and bottom strand oligonucleotides. Adapter bottom strand oligonucleotide is 5′ phosphorylated and 3′ blocked to prevent adapter self-ligation. DNA library for Illumina next-generation sequencing (NGS) is made by PCR amplification using Illumina primers corresponding to adapter sequences. Paired-end sequencing is performed using standard read1 and read2 primers. (C) 5′ adapter ligation method2. First-strand cDNA primed using RTCIRC (Table S1) with 5′ phosphate ligation donor group (5′-PO_4_) is intra-molecularly ligated using CircLigase 2 enzyme. The 3′ tag sequence in RTCIRC contains both 5′ and 3′ adapter sequences (depicted in dark purple and magenta, respectively). DNA library for NGS is made by PCR amplification and paired-end sequenced as for method1.

We present a new method for identifying 5′ capped nucleotides from mRNA transcripts. mRNA is purified from the biological sample and used to synthesize cDNA. A portion of the cDNA is reserved as unenriched for generation of a control library and the remainder is enriched for full-length cDNA (Fig. 1A). Full-length cDNA fragments are enriched using recombinant eIF4E protein, which is capable of binding to the 5′ cap nucleotide in first-strand cDNA products (Edery et al., 1995). We enriched full-length cDNA using recombinant *P*. *falciparum* eIF4E protein, which has an affinity for the 5′ cap nucleotide similar to mammalian eIF4E (Shaw et al., 2007). To mitigate 5′ end biases when adding adapter, we employ two different adapter ligation protocols, namely cDNA tailing followed by double-stranded adapter ligation (adapter ligation method1) and intramolecular ligation (adapter ligation method2) (Figs. 1B, 1C). 5′ end bias patterns were observed among the unaligned reads, in which C was markedly lower for the first base of method1 HiSeq libraries compared with downstream bases (Fig. S1), and G markedly lower for the first base of all method2 libraries (Figs. S1 and S2).

### Accuracy and sensitivity of the method

**Figure 2:**
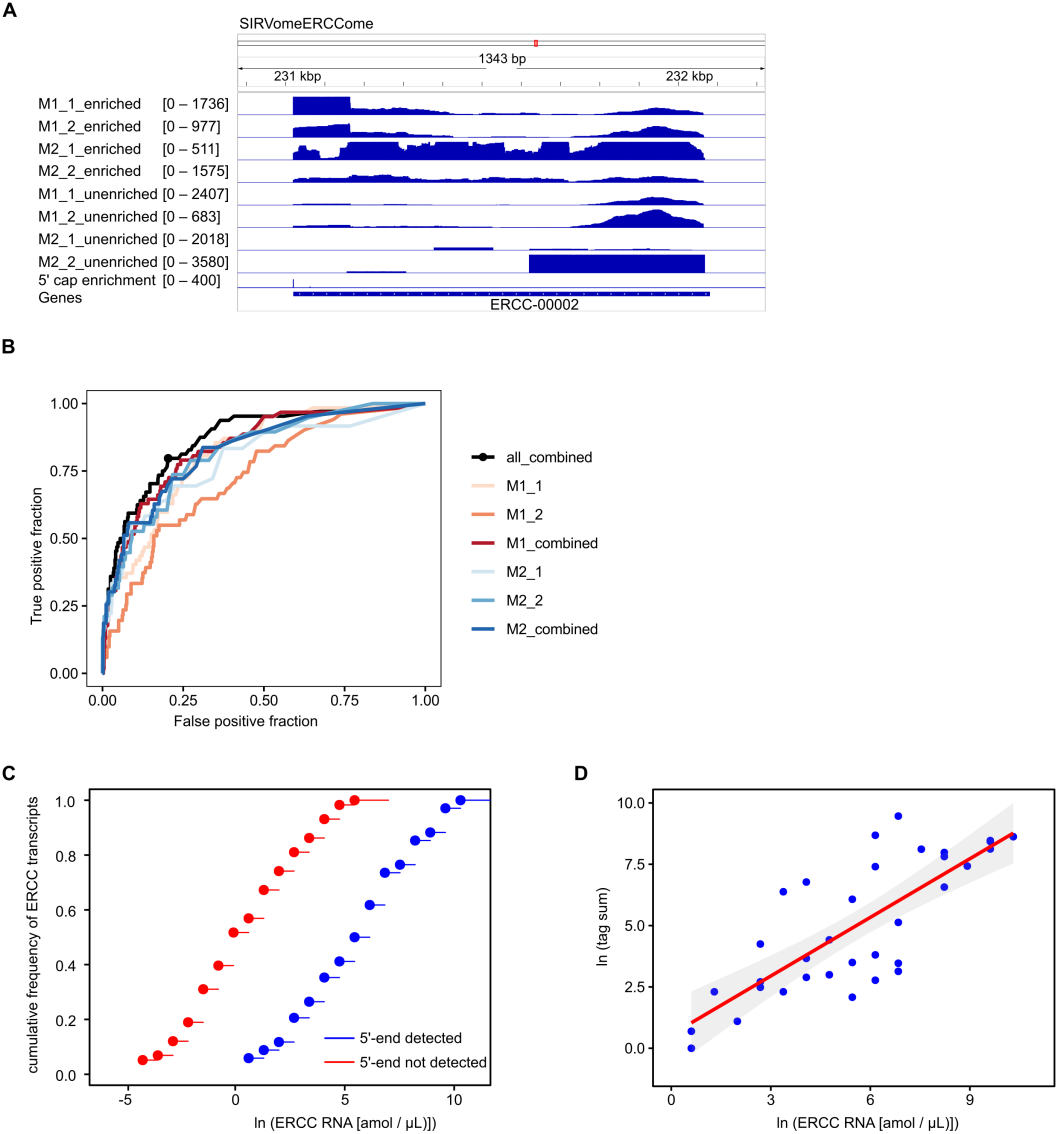
Validation of 5′ cap enrichment using synthetic RNA. *Plasmodium falciparum* mRNA was spiked with SIRV set 3 (a pool of synthetic RNAs with known 5′ capped ends). Two experiments were conducted using 5′ adapter ligation method1 (M1_1 and M1_2) and two with 5′ adapter ligation method2 (M2_1 and M2_2). To test whether the power to detect was increased by combining enrichment *P*-values from different experiments, combined *P*-values were calculated from M1_1 and M1_2 experiments (M1_combined), M2_1 and M2_2 experiments (M2_combined) and all four experiments (all_combined). Enrichment *P*-values were transformed to integer counts and clusters of enrichment signals were identified by analysis with CAGEr (Haberle et al., 2015). CAGEr cluster signals were used for further analyses. (A) Example of transcriptomic data for the ERCC-00002 synthetic RNA. Reads from enriched and unenriched libraries were aligned to the reference sequence (SIRvomeERCCome). Data tracks underneath the chromosome line show normalized read depth for each library, with the range of depth values in the interval shown indicated in brackets. The bottom data track shows the integer counts of 5′ capped nucleotide enrichment from four experiments (5′ cap enrichment) with the range of integer counts in the interval shown indicated in the bracket. Figure produced using IGV software (Robinson et al., 2011). (B) Receiver Operator Characteristic (ROC) plots. The black circle on the “all_combined” plot represents the optimal cut-point of enrichment signal (CAGEr cluster tag sum) equal to 32. (C) Empirical cumulative distribution frequency plots of natural log RNA concentration of External RNA Controls Consortium (ERCC) synthetic RNAs. The ERCC RNAs are the subset of synthetic RNAs with a single transcript annotated for each *in silico* gene. The 5′ end was detected for 34 ERCC RNAs, whereas the 5′ end was not detected for 58 ERCC RNAs. The distributions of the two groups were compared by two-sample Kolmogorov–Smirnov test (*P*-value = 1.142e^−07^). (D) Correlation of natural log (CAGEr cluster tag sums) with natural log concentration of ERCC synthetic RNAs with detected 5′ end. The red line represents linear regression of the data, *P* = 3.941e^−08^. 95% confidence intervals are indicated by the gray bands.

To assess the accuracy and sensitivity of the proposed method, we performed four experiments with *P*. *falciparum* mRNA spiked with 5′ capped synthetic RNAs, including two replicates for each adapter ligation protocol. The reads mapped to the *in silico* SIRVomeERCCome reference sequence were used to determine enrichment signals for synthetic RNAs with known 5′ ends (Fig. 2A). The performance for detecting 5′ capped ends was assessed by Receiver Operator Characteristic (ROC) plot (Fig. 2B). As the same batch of synthetic RNA was used for the four spike-in experiments, we also tested whether combining *P*-values from replicate experiments could improve performance. Individual experiments with method2 gave slightly higher performance than method1 as shown by the greater area under ROC curves. The greatest performance was achieved by combining *P*-values from all four experiments. From the ROC curve of all four experiments combined, the optimal cutpoint for classifying 5′ capped nucleotide signals was determined (Fig. 2B). We assessed the sensitivity of the method for detecting 5′ capped ends in absolute terms by comparing the distributions of the ERCC spike-in RNA concentrations for detected *versus* undetected 5′ ends, which were significantly different (Fig. 2C). Moreover, the 5′ capped nucleotide signals for ERCC spike-in RNAs showed a significant positive correlation with the known RNA concentrations indicating that the enrichment signals are quantitative and reflect RNA abundance (Fig. 2D). From the analysis of spike-in RNA enrichment data, we determined the performance of the method using different adapter-ligation protocols and showed that combining *P*-values from all available replicate experiments gives the best performance.

### Annotation of 5′ capped nucleotides in *P*. *falciparum*

**Figure 3:**
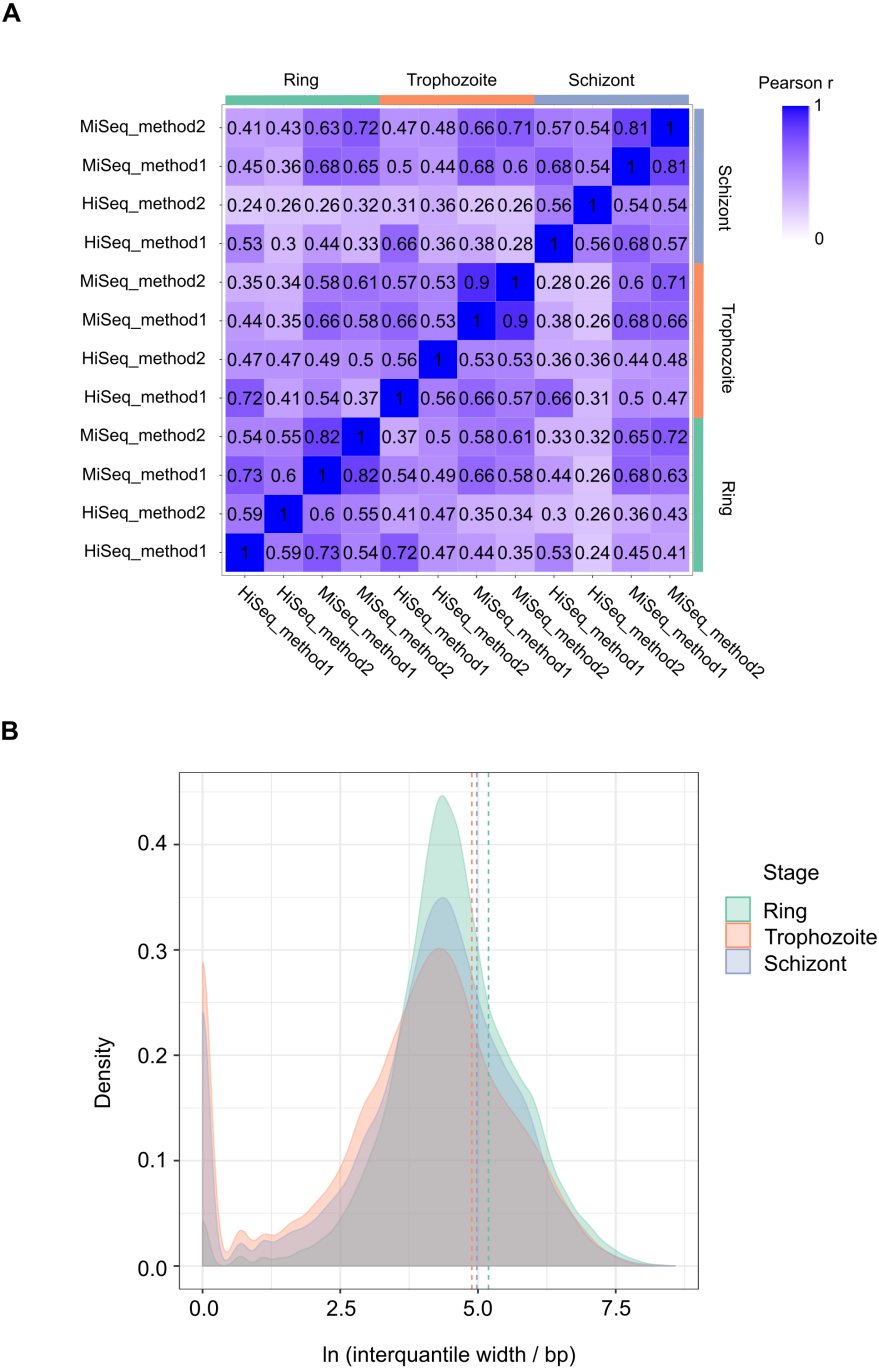
Transcriptomic data and corresponding 5′ capped nucleotide signals in *Plasmodium falciparum*. Twelve transcriptomic experiments were conducted for ring, trophozoite and schizont stages of *P. falciparum* development. Sequence data were obtained for six experiments using the Illumina HiSeq platform and six with the Illumina MiSeq platform. Sequencing libraries were generated using adapter ligation method1 and method2 for each sampled stage and sequencing platform. Library pairs of unenriched control and 5′ cap enriched were generated for each experiment. (A) Pairwise correlations of experimental data. Expressions of annotated genes were determined from unenriched control library data and used to determine pairwise Pearson’s correlation. Samples from the same stage of development are grouped as shown by the bars next to the correlation matrix. The sequencing platform used is indicated by the prefix (HiSeq or MiSeq) and the adapter ligation protocol by the suffix (method1 or method2). The Pearson r value for each combination is shown in the matrix and colored according to the scale shown on the right. (B) Distributions of interquantile cluster widths of 5′ capped nucleotide signals. 5′ cap enrichment was determined for all transcribed nucleotides from the paired transcriptomic data using statistical models. The combined enrichment *P*-values of four replicate experiments for each development stage were transformed to integer counts and clustered using CAGEr (Haberle et al., 2015). The interquantile width distribution of 5′ capped nucleotide signals passing threshold (CAGEr cluster tpm > 32) is shown by kernel density plot fitted to natural log of cluster width (bp). The dashed lines indicate the mean cluster width for each stage (green, ring 180 bp; orange, trophozoite 133 bp; blue, schizont 145 bp).

We performed 12 experiments in total for identifying 5′ capped ends in *P*. *falciparum* mRNA isolated from different stages of the IDC. Six experiments were conducted with high sequence coverage on the HiSeq platform (including the four with synthetic RNA spike-in) and six at lower coverage on the MiSeq platform. To assess variability among samples, gene expression profiles were compared. All pairwise combinations of samples from the same stage of the IDC (including samples prepared using different library and sequencing protocols) showed Pearson r values greater than 0.5, whereas some pairwise combinations of samples from different stages showed Pearson r less than 0.5 (Fig. 3A). Enrichment *P*-values were thus combined from four replicate experiments of each stage of development to increase the power of detection. 5′ capped nucleotide signals were annotated for ring, trophozoite and schizont stages of *P*. *falciparum* IDC based on the threshold of 5′ capped nucleotide signal determined from analysis of spike-in RNA (Table S4). The 5′ capped nucleotide signals detected for *P*. *falciparum* encompass genomic regions of variable width (Table S4). In other transcriptomic studies of eukaryotic 5′ capped nucleotides, the majority of signals correspond with core promoters, or genomic regions of varying width in which multiple transcription start sites (TSS) are used at different frequencies (Zhao et al., 2011). The interquantile signal width reported by CAGEr can be used for the analysis of 5′ capped nucleotide distribution (Haberle et al., 2015). Bimodal distributions comprising sharp (<10 bp) and broad (>10 bp) peaks were observed for each sampled stage of the *P*. *falciparum* IDC; however, the majority of signals showed broad peak widths (Fig. 3B). Many of the 5′ capped nucleotide signals from the three stages of development overlapped the same genomic regions, which made it difficult to separate signals of alternative 5′ ends used dynamically throughout the IDC. Therefore, to simplify the genomic analysis we collapsed the signals across different stages into 17,961 non-overlapping intervals. The dominant 5′ capped nucleotide (5CN) with the strongest signal across the IDC in each genomic interval was investigated in more detail.

### Sequence patterns in the vicinity of dominant 5′ capped nucleotides

**Figure 4:**
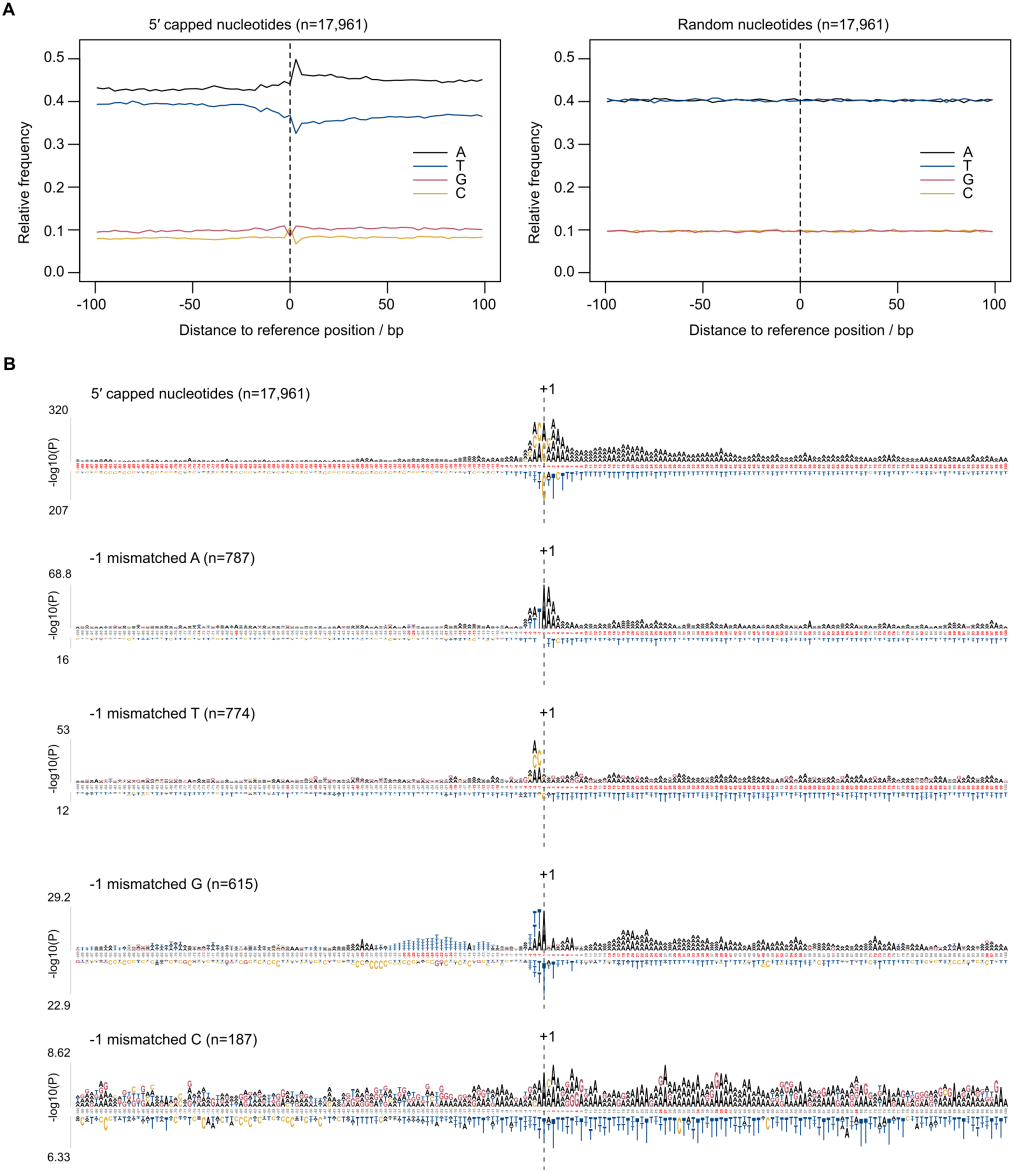
Sequence patterns in the vicinity of 5′ capped nucleotides. *Plasmodium falciparum* 3D7 genomic sequences flanking 17,961 dominant 5′ capped nucleotides (5CN) annotated from 5′ cap enriched transcriptomic data and 17,961 randomly selected positions were used for sequence analysis. (A) Average base-composition plots in the vicinity of 5CN (left) and random positions (right). Plots were generated using the seqPattern package in R. (B) Position-specific patterns of 1–4 nt k-mers in the vicinity of 5CN identified by kpLogo (Wu & Bartel, 2017) using random genomic sequences as background. The height of each k-mer symbol represents the –log_10_*P-* value from statistical testing. Over-represented k-mers are shown above the number line and under-represented below. Bases in the number line highlighted in red indicate positions with Bonferroni-corrected significant k-mers. Sequences flanking all 17,961 5CN were analysed (top) and the subset of sequences with high frequencies ( >50%) of aligned reads starting with a mismatched base 1 bp upstream (−1 position) of 5′ capped nucleotides. Patterns are shown for sequences with mismatched A, T, C and G reads at the −1 position.

Eukaryotic promoter regions are characterized by A/T richness and the presence of position-specific short motifs such as upstream TATA boxes and pyrimidine/purine at the −1/ + 1 position (where +1 denotes TSS) (Müller & Tora, 2014). To test whether TSS sequence patterns existed at 5CN, base compositions were investigated. The average base composition at 17,961 5CN and 100 flanking genomic bases showed elevated A composition, in particular at +1 and positions immediately downstream compared with random (Fig. 4A).

Aligned reads may possess non-reference (mismatched) 5′ terminal nucleotides that can affect the accuracy of annotating transcript 5′ ends. For example, analysis of CAGE data requires correction for the non-reference G present on the first base of the majority of aligned reads, which originates from the cap signature (Haberle et al., 2015). The odds ratio of mismatched base counts from read1 starts aligned one base upstream of 5CN (−1 position) was compared with all other transcribed positions. Odds ratios significantly greater than 1 were observed for mismatched A(8/12 datasets), C(2/12 datasets), G(7/12 datasets) and T(1/12 datasets) (Table S3). Although mismatched bases are more common at −1 than at other transcribed positions, it should be noted that the majority of aligned reads do not start with a mismatch (Table S3). To test whether base mismatching of aligned reads could be influenced by reference sequence, sequence patterns were investigated among genomic regions flanking 5CN with high frequencies (>50%) of aligned reads with mismatched first bases (Fig. 4B). Among genomic regions in the vicinity of all 5CN, we observed significant over-representation of A-rich motifs at the 5CN (+1) and downstream positions. CA was the most significant over-represented motif at the −1 position. Different over-represented motifs were identified among genomic regions with high frequencies of aligned reads with mismatches at the -1 position. T, AA, TA and CA were identified as the most significant over-represented motifs among sequences with high frequencies of non-reference A, C, G and T at -1 positions, respectively.

### Genomic features in the vicinity of dominant 5′ capped nucleotides

**Figure 5:**
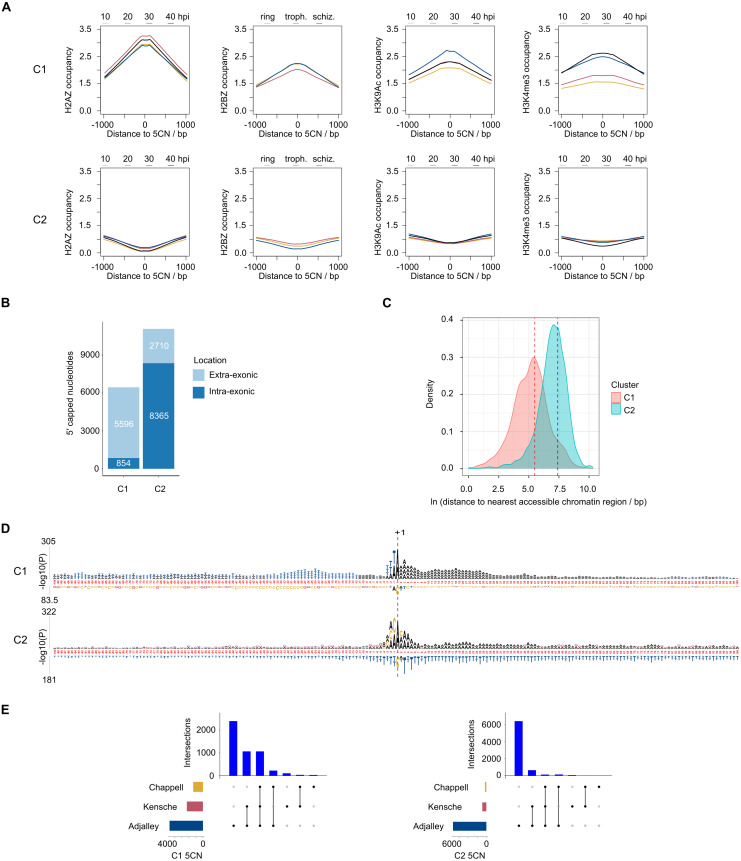
Clustering of *Plasmodium falciparum* 5′ capped nucleotides by genomic features. Genomic features were analyzed for dominant 5′ capped nucleotides (5CN) annotated from 5′ cap enriched transcriptomic data. Two clusters of 17,525 5CN (C1 and C2) were resolved by unsupervised clustering (Fig. S3) using chromatin-immunoprecipitation sequencing (ChIP-seq) data of H2AZ, H2BZ, H3K9ac and H3K4me3 occupancies (Bártfai et al., 2010; Hoeijmakers et al., 2013). (A) Epigenetic marks in the vicinity of 5CN. Score matrices were constructed from ChIP-seq data for each 5CN in the genome and 1,000 bp flanks, and plots of average scores for cluster C1 (top) and C2 (bottom) made using the genomation package (Akalin et al., 2015) in R. Data from different stages of the intra-erythrocytic development cycle were plotted on the same axes for each type of chromatin feature shown. Numbering on the *y*-axes refers to average normalized occupancies. (B) Compositions of cluster C1 and C2 5CN with respect to annotated protein-coding exons. (C) Distributions of 5CN distance to nearest accessible chromatin region (annotated peak signal from Assay for Transposase-Accessible Chromatin sequencing data (Toenhake et al., 2018; Ruiz et al., 2018; Yin et al., 2020) are shown by kernel density plot fitted to natural log of distance (bp). The mean distance is shown by the dashed line for each distribution (C1, 243 bp; C2, 1,641 bp).(D) Position-specific patterns of 1–4 nt k-mers in the vicinity of 5CN identified by kpLogo (Wu & Bartel, 2017) using 17,961 random genomic sequences as background. The height of each k-mer symbol represents the –log_10_*P-* value from statistical testing. Over-represented k-mers are shown above the number line and under-represented below. Bases in the number line highlighted in red indicate positions with Bonferroni-corrected significant k-mers.(E) Intersection of 5CN with 5′ capped nucleotide enrichment data from independent studies. Intersection of 5CN with 5′ cap nucleotide enrichment data from 5′ cap sequencing (Adjalley et al., 2016), and SMART enrichment (Kensche et al., 2016; Chappell et al., 2020) was scored if read depth was two or greater. Intersections are shown by UpSetR plot (Conway, Lex & Gehlenborg, 2017), which is a matrix-based representation of set sizes and their intersections. Matrix rows represent the sets (5CN annotated from data in this study) and columns represent intersections of 5CN supported by data in other studies. Set sizes are shown by the horizontal bar plots on the left. All combinations of set intersections are shown by the matrix cell on the right, in which sets that are part of a given intersection are represented by black-filled circles. Sets that are not part of the intersection are shown as light gray circles. The sets considered in each intersection (black circles) are connected by vertical black lines to emphasize the column-based relationships. Bars above the matrix columns represent the number of 5CN in each intersection. Intersections of 5CN with other studies are shown separately for C1 (left) and C2 (right).

9,334 (52%) of the 5CN are located within *P*. *falciparum* protein-coding exons (Table S4). Given that eukaryotic TSS are typically located outside of protein-coding regions, many of the exonic 5CN may represent transcriptional noise unrelated to TSS. We hypothesized that epigenetic information could be used to classify TSS in a manner naïve to gene annotation. Eukaryotic core promoter regions possess a distinctive chromatin architecture, characterized by high occupancy of nucleosomes with variant histone H2AZ, and histone modifications including H3K9 acetylation and H3K4 trimethylation (Jiang & Pugh, 2009; Müller & Tora, 2014). We used published *P*. *falciparum* epigenetic data of H2A.Z, H3K9 acetylation and H3K4 trimethylation (Bártfai et al., 2010) and H2B.Z (Hoeijmakers et al., 2013) for unsupervised clustering of 5CN. H2B.Z is an apicomplexan-specific histone variant that colocalizes with H2A.Z (Hoeijmakers et al., 2013; Petter et al., 2013). The *P*-values from unimodal tests were below the threshold of significance (Silverman critical bandwidth test *P* = 0.01; Hall and York critical bandwidth test *P* < 2.2e^−^^16^; Ameijeiras-Alonso et al. excess mass test *P* < 2.2e^−^^16^; Cheng and Hall excess mass test *P* < 2.2e^−16^; Fisher and Marron Cramer-von Mises test *P* < 2.2e^−^^16^; Hartigan and Hartigan dip test *P* < 2.2e^−^^16^). Hence, we rejected the null hypothesis that the data are unimodal and conclude that more than one cluster exists. Two clusters of 5CN (C1, 6,450 positions; C2, 11,075 positions) were resolved by unsupervised clustering (Fig. S3; Table S4). Furthermore, the number of relevant clusters by the majority vote of clustering indices was two (Fig. S3). C1 5CN overlap genomic regions with locally high occupancies of H2AZ, H2BZ, H3K9 acetylation and H3K4 trimethylation chromatin marks across the IDC, whereas C2 5CN show low occpancies of these chromatin marks. (Fig. 5A). C1 5CN overlap high occupancies of other histone acetylation and methylation marks, with the notable exception of H3K4me1, which is elevated among C2 5CN (Fig. S4). C1 and C2 5CN are distinguished by other epigenetic features, including elevated nucleosome, PfGCN5 histone acetyltransferase and RNApolII occupancies for C1 (Fig. S5). A higher proportion of 5CN are located outside of protein-coding exons for C1 than C2 (Fig. 5B), and the average distance to the nearest accessible chromatin feature is less for C1 than C2 (Fig. 5C). Analysis of genomic sequence in the vicinity of 5CN showed different patterns of motifs among C1 and C2 (Fig. 5D). T-rich motifs are over-represented upstream of C1 5CN, whereas they are under-represented in the vicinity of C2. The most significant over-represented motifs at the −1 position are TA and CA for C1 and C2, respectively. Previous studies of transcript 5′ ends in *P*. *falciparum* highlighted patterns of genomic features similar to the C1 group, including elevated H2AZ, H3K9 acetylation and H3K4 trimethylation chromatin marks and high local A/T contents (Adjalley et al., 2016; Kensche et al., 2016; Chappell et al., 2020) Correspondingly, a greater proportion of 5CN in the C1 group than the C2 group are supported by data in the other studies (Fig. 5E; Table S4).

### Patterns of 5′ capped nucleotides among genes and associated transcripts

**Figure 6:**
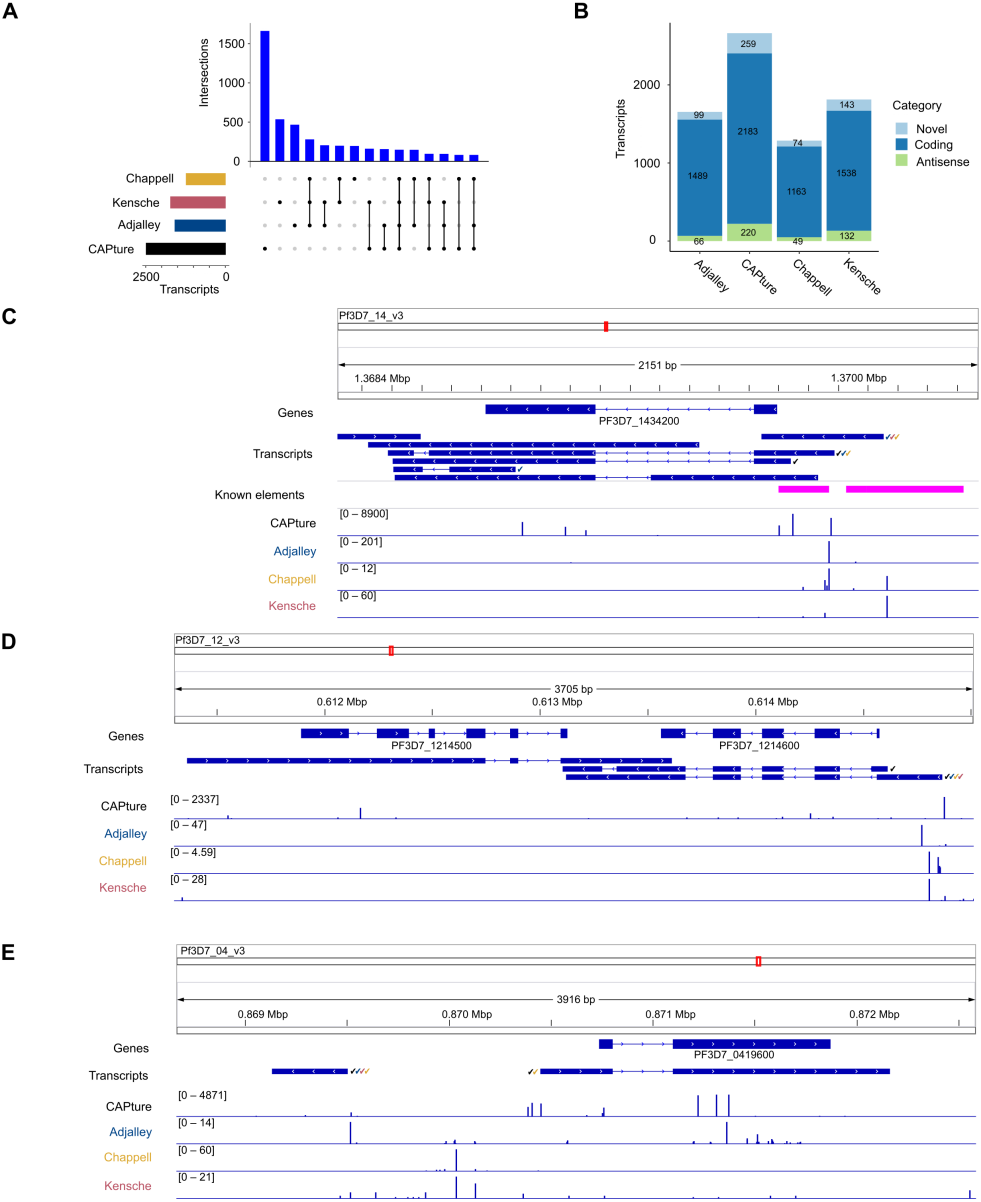
Annotation of 5′ capped transcripts in *Plasmodium falciparum*. *P. falciparum* transcripts were annotated from FLAIR analysis (Tang et al., 2020a) of direct RNA sequencing data (Lee et al., 2021). 5′ capped transcripts were identified by the overlap of 5′ end nucleotide position with 5′ capped nucleotide enrichment data. (A) Intersection of transcript 5′ ends with 5′ capped nucleotide enrichment data from independent studies. Intersections of transcript 5′ ends are shown with data from this study (labeled CAPture), 5′ cap sequencing ((Adjalley et al., 2016), labeled Adjalley), and SMART enrichment ((Kensche et al., 2016), labeled Kensche; (Chappell et al., 2020), labeled Chappell). Intersections are shown by UpSetR plot (Conway, Lex & Gehlenborg, 2017), which is a matrix-based representation of set sizes and their intersections. Matrix rows represent the sets (transcripts) and columns represent intersections of transcript 5′ ends with 5′ cap nucleotide positions annotated from each study. Set sizes are shown by the horizontal bar plots on the left. All combinations of set intersections are shown by the matrix cell on the right, in which sets that are part of a given intersection are represented by black-filled circles. Sets that are not part of the intersection are shown as light gray circles. The sets considered in each intersection (black circles) are connected by vertical black lines to emphasize the column-based relationships. Bars above the matrix columns represent the number of transcripts in each intersection.(B) Categories of 5′ capped transcripts supported by 5′ capped nucleotide enrichment data from different studies. The numbers of transcripts in each category (coding, antisense, and novel) are indicated by stacked barplots for each 5′ capped nucleotide enrichment study. Parts C –E show examples of 5′ capped transcripts in genomic context. Figures were generated using IGV software (Robinson et al., 2011). Annotated genes, including the boundaries of coding exons (blue bars) and introns (blue lines) are shown in the Genes tracks. Transcripts annotated by FLAIR are shown in the Transcripts tracks. The signals of dominant 5′ capped nucleotides from full-length cDNA enrichment studies are indicated in the tracks labeled CAPture (this study), Adjalley (5′ cap sequencing reported by (Adjalley et al., 2016), Chappell (5UTR-seq reported by (Chappell et al., 2020) and Kensche (SMART enrichment reported by (Kensche et al., 2016)). The 5′ capped nucleotide signals are shown as values of tpm.dominant_ctss reported by combined CAGEr (Haberle et al., 2015) analysis of data across intraerythrocytic stages of *P*. *falciparum* development with the range values in the interval shown indicated in the bracket in each track. Full-length transcripts with 5′ ends that correspond to dominant 5′ capped nucleotides are indicated by tick marks next to the transcript, which are colored according to which full-length cDNA enrichment study is in agreement (CAPture, black; Adjalley, blue; Chappell, yellow; Kensche, red). (C) PF3D7_1434200 (calmodulin) gene region. Known elements are indicated by magenta boxes, including 5′ ends mapped by 5′ rapid amplification of cDNA ends (5′-RACE) (Polson & Blackman, 2005) from the most proximal to the most distal and upstream promoter/enhancer element mapped by reporter gene analysis (Crabb & Cowman, 1996). (D) Example of antisense transcripts for the PF3D7_1214500 gene, which are also coding transcripts for the adjacent PF3D7_1214600 gene. (E) Example of a novel transcript upstream of the PF3D7_0419600 gene.

The transcriptomic surveys from data generated in this study and others (Adjalley et al., 2016; Kensche et al., 2016; Chappell et al., 2020) provide comprehensive information of transcript 5′ capped ends. In order to annotate transcripts, data of complete (end-to-end) transcript structures are required, which can be difficult to obtain from short-read cDNA data owing to breaks in coverage at highly AT-rich or unmappable regions of the *P*. *falciparum* genome (Chappell et al., 2020). To our knowledge, the largest available dataset of complete transcript structures was obtained by Nanopore long-read direct RNA sequencing (Lee et al., 2021). In direct RNA sequencing, the transcript is sequenced from the 3′ end. However, the 5′ cap modification cannot be identified from the data as a variant base call, and it is therefore not possible to determine if the transcript sequence is complete (extends to the original transcript origin) or not. Transcripts inferred from direct RNA sequencing are often truncated because of RNA degradation, electronic noise generated during the sequencing process and low signals near to the 5′ end (Soneson et al., 2019; Workman et al., 2019). We created *P*. *falciparum* transcript annotations by FLAIR analysis of the direct RNA sequencing data (Data S1). Full-length transcripts were identified from the correspondence of transcript 5′ end locations with 5CN from cDNA data (Table S5; Fig. 6). A total of 4,512 transcript 5′ ends (42%) corresponded with 5CN from one or more studies. The greatest number of transcripts annotated by FLAIR corresponded with 5CN from our data (2482), followed by 5CN from (Kensche et al., 2016; Adjalley et al., 2016; Chappell et al., 2020) with 1,720, 1,580, and 1,230 transcripts, respectively. Some transcript 5′ ends corresponded with 5CN from more than one study, but only 148 transcript 5′ ends corresponded with 5CN from all four studies (Fig. 6A). The majority of full-length transcripts overlap annotated genes/coding regions, with minorities of antisense and novel transcripts (Fig. 6B). Multiple full-length transcript isoforms with alternative 5′ ends are apparent for *P*. *falciparum* genes, including the PF3D7_1434200 (calmodulin) gene shown in Fig. 6C. The 5′ ends of two full-length coding transcripts map to a transcription initiation region upstream of the calmodulin gene mapped previously by RNA-ligase mediated 5′ rapid amplification of cDNA ends (Polson & Blackman, 2005). The untranslated regions of *P*. *falciparum* gene transcripts can extend for several hundred bases beyond the terminal coding exons (Chappell et al., 2020), such that the transcripts can overlap coding exons of adjacent genes. In the example shown in Fig. 6D, the 3′ untranslated region of a full-length coding transcript for the PF3D7_1214600 gene overlaps, and is antisense to the adjacent PF3D7_1214500 gene. Although the *P*. *falciparum* genome is gene-dense with median intergenic distance less than 2 kb (Russell et al., 2013), novel full-length transcripts not overlapping coding exons are present. In the example shown in Fig. 6E, a novel transcript initiates near to a full-length coding transcript of the PF3D7_0419600 gene on the opposite strand.

## Discussion

Accurate annotation of eukaryotic genes, in particular those of less well-characterized organisms such as *P*. *falciparum,* requires detailed transcript information. A major challenge for transcript annotation is identifying 5′ ends because truncated RNA or cDNA sequences are common experimental artifacts in transcriptomic data. Previous transcriptomic studies of *P*. *falciparum* employing methods for enrichment of full-length cDNA were not comprehensive as transcript 5′ ends could not be detected for some genes known to be expressed in the IDC (Adjalley et al., 2016; Kensche et al., 2016; Chappell et al., 2020). To provide a more complete catalog of transcript 5′ ends in *P*. *falciparum*, we developed a novel method to complement the available data. In our method, full-length cDNA is enriched using 5′ cap binding protein eIF4E (Edery et al., 1995). Instead of eIF4E protein, other agents with selective 5′ cap binding affinity can be used for enriching 5′ capped RNA (Bhardwaj et al., 2019). To separate enriched 5′ end signals from noise, the unenriched background within the body of each transcript can be modeled from local windows assumed to represent the same transcript (Bhardwaj et al., 2019). This data analysis method though is not likely to be effective in organisms such as *P*. *falciparum* with gene-dense genomes, in which multiple overlapping transcripts of varying abundance can arise from the same genomic region (Van Lin et al., 2001). In our method, the unenriched cDNA is sequenced in parallel as a control, which allows for statistical modeling of enrichment at the nucleotide level. Moreover, the power to detect 5′ end signals can be increased by combining enrichment *P*-values from replicate experiments as shown using spike-in RNA (Fig. 2). Although performance is increased with more replicates, the accuracy of the method is limited by the low efficiency of biochemical enrichment of full-length cDNA, as the eIF4E protein used for enrichment has micromolar affinity for the mRNA 5′ cap (Shaw et al., 2007).

The processes of adding sequencing adapter in oligo-capping and SMART are integral to the process of enrichment for full-length cDNA. Therefore, it is not possible to obtain a matching unenriched cDNA control for statistical modelling with these methods. We employed two different protocols for adding sequencing adapter to cDNA ends which retain transcript 5′ terminal nucleotides and can be used with unenriched and full-length enriched cDNA. However, the short homopolymer tail at the beginning of read1 from method1 libraries can be problematic for some NGS platforms. We circumvented the problem of low diversity caused by the homopolymer tail for method1 libraries by sequencing in one lane of a flow cell shared with more diverse libraries in other lanes (HiSeq platform) or using a custom dark-cycle sequencing recipe (MiSeq platform). The observed lower frequency of C for the first base of method1 HiSeq libraries compared with downstream bases (Fig. S1) is a consequence of the bioinformatic trimming of the homopolymer tail appended to the cDNA end. If the tail is not trimmed, read alignment accuracy could be affected because of mismatches to the reference. The observed lower frequency of G for the first base of method2 libraries compared with downstream bases (Figs. S1 and S2) is consistent with the reported Circligase ligation bias (Kwok et al., 2013) against the complementary C ligation acceptor on first-strand cDNA in method2. The different 5′ end biases for method1 and method2 could limit detection of certain 5′ capped nucleotides, although if both protocols are used they can complement each other (Fig. 2).

A bimodal distribution of transcript 5′ end signal was observed from our data, suggesting the presence of sharp and broad 5′ end signals in *P*. *falciparum*. Sharp and broad 5′ end signals were also described in other *P*. *falciparum* studies (Chappell et al., 2020; Adjalley et al., 2016), although the width distribution inferred from the data depends on the noise filtering and clustering parameters employed. Locally high A contents were observed at 5CN (Fig. 4A), in agreement with the patterns of *P*. *falciparum* transcript 5′ ends described in other studies (Chappell et al., 2020; Adjalley et al., 2016; Kensche et al., 2016). Aligned reads starting with mismatched bases are significantly more prevalent at the -1 position in our data than other transcribed regions (Fig. 4B). Because of the locally biased base content in the vicinity of 5CN, reads with mismatched T or A may arise from technical errors in these regions, *e.g.*, template slippage during reverse-transcription, PCR, or sequencing by synthesis reactions. For -1 positions with high frequencies of reads starting with mismatched G, the over-represented TA motif suggests a biological reason, most likely the cap signature of reverse-transcribed 5′ cap m^7^G.

Two groups of 5CN in *P*. *falciparum* (C1 and C2) were identified by unsupervised clustering of our data. Although determining the mechanisms that generate the different types of 5CN is beyond the scope of the present study, the genomic contexts of 5CN provide clues as to their origins. C1 likely represents TSS since they overlap genomic regions with high local occupancies of H2AZ, H3K9Ac and H3K4me3 histone modifications (Fig. 5A, Figs. S4, S5) that are strongly associated with transcription initiation in other eukaryotes (Müller & Tora, 2014). Other evidence to support the conclusion that C1 represents TSS includes the following epigenetic patterns consistent with TSS in other eukaryotes: (i) elevated occupancies of other histone acetylation marks (H3K14Ac, H3K18Ac, H3K27Ac; Fig. S4) that are associated with eukaryotic transcriptional activation (Zhao & Garcia, 2015), (ii) patterns of elevated occupancy of H3K4me2, but reduced occupancy of H3K4me1 (Fig. S4) and reduced occupancy of H3 (Fig. S5) that are associated with TSS (Koch et al., 2007), (iii) elevated nucleosome and RNApolII occupancies (Fig. S5) that are associated with TSS (Zhao et al., 2011), and (iv) elevated occupancy of PfGCN5 (Fig. S5), the *P*. *falciparum* homolog of GCN5 protein (Bhowmick et al., 2020), which is a component of the SAGA transcription coactivator complex that acetylates histones at core promoters and enhancers (Cheon et al., 2020). Furthermore, C1 nucleotides are located mostly outside of protein-coding exons (Fig. 5B) and are closer to accessible chromatin (Fig. 5C) where transcription is more likely to initiate. The TA over-represented motif at the -1 position for the C1 group (Fig. 5D) is consistent with a eukaryotic transcription initiation motif (Müller & Tora, 2014), and the upstream T-rich and downstream A-rich motifs are consistent with eukaryotic TSS locator motifs (Lubliner, Keren & Segal, 2013).

The C2 group of 5CN lacks epigenetic features of TSS (Fig. 5A). C2 nucleotides are also more prevalent in coding regions (Fig. 5B) and are further away from accessible chromatin (Fig. 5C) where transcription is less likely to initiate. C2 nucleotides are less well supported by other methods (Fig. 5E; Table S4) and many could therefore represent technical noise. However, the significant over-representation of sequence motifs including CA at the -1 position (Fig. 5D) is indicative of a biological phenomenon that requires further investigation. C2 nucleotides show elevated occupancy of H3K4me1 (Fig. S4), which is elevated distal to TSS in eukaryotes (Koch et al., 2007; Zhao et al., 2011). High occupancy of H3K4me1 is observed at the start sites of enhancer RNAs (eRNAs) in eukaryotes, which represent a class of generally low-abundance non-coding RNA; C2 nucleotides are however unlikely to represent eRNA start sites, as they generally do not overlap genomic regions associated with accessible chromatin and the H3K27Ac chromatin mark characteristic of eRNA start sites (Lewis, Li & Franco, 2019). Rather than TSS or 5′ ends of eRNAs, C2 could represent transcript 5′ ends generated post-transcriptionally by co-translational cleavage and cytoplasmic recapping (Trotman & Schoenberg, 2019). This is speculative though since it is not known if mRNA capping occurs post-transcriptionally in the *P*. *falciparum* cytoplasm. However, co-translational cleavage could occur as truncated transcript isoforms are evident in *P*. *falciparum* polysomal fractions (Bunnik et al., 2013).

The 5′ ends of 4,512 transcripts annotated from direct RNA sequencing corresponded to 5CN from cDNA data, suggesting that these transcripts are 5′ capped. However, the lack of corroborative cDNA data for the majority of transcripts from direct RNA sequencing suggests that they are truncated. It should be noted though that relatively few annotated transcript 5′ ends correspond to 5CN from more than one study (Fig. 6A), suggesting that verification of transcript 5′ capped ends from short-read cDNA sequencing data is challenging, perhaps because of dynamic usage of transcript initiation sites during development (Adjalley et al., 2016; Chappell et al., 2020), different 5′ end biases among full-length cDNA enrichment methods (see above), stochastic variation in TSS usage (Xu, Park & Zhang, 2019), and the difficulty in aligning short cDNA reads to extremely AT-rich regions in the *P*. *falciparum* genome (Chappell et al., 2020). Although the majority of *P*. *falciparum* transcripts overlap coding regions (Fig. 6B), many transcripts with alternative 5′ capped ends partially overlap the annotated open reading frame (Fig. 6C, Fig. 6D). These alternative transcripts could possess functions for production of N-terminal truncated protein isoforms, or regulatory non-coding RNA (Trotman & Schoenberg, 2019). On the other hand, transcripts with alternative 5′ capped ends could represent RNA decay intermediates (Arribere & Gilbert, 2013).

## Conclusions

A new transcriptomic approach for identifying mRNA 5′ capped nucleotides was developed to comprehensively annotate *P*. *falciparum* 5′ capped transcripts expressed in intraerythrocytic stages of the life cycle. Two groups of 5CN were annotated from data generated using the new method with distinctive epigenetic and genomic sequence patterns. The correspondence of 5CN with transcript 5′ ends inferred from direct RNA sequencing revealed patterns of 5′ capped transcripts, including the widespread occurrence of transcripts with alternative 5′ ends that partially overlap gene coding regions.

## Supplemental Information

10.7717/peerj.11983/supp-1 Supplemental Information 1 Base composition plots of read1 from libraries sequenced using the HiSeq platform Plots of average base composition (A, green; T, red; C, blue, G, black) at different positions of read1 were generated using FastQC (Andrews, 2010). The .fastq raw files were preprocessed with Cutadapt 1.18 (Martin, 2011) to remove the homopolymer tail added to cDNA for adapter ligation .
Andrews S. 2010. FastQC: a quality control tool for high throughput sequence data. Available at http://www.bioinformatics.babraham.ac.uk/projects/fastqc (accessed on 6 May 2021)

10.7717/peerj.11983/supp-2 Supplemental Information 2 Base composition plots of read1 from libraries sequenced using the MiSeq platform Plots of average base composition (A, green; T, red; C, blue, G, black) at different positions of read1 were generated from raw .fastq files using FastQC (Andrews, 2010).
Andrews S. 2010. FastQC: a quality control tool for high throughput sequence data. Available at http://www.bioinformatics.babraham.ac.uk/projects/fastqc (accessed on 6 May 2021)

10.7717/peerj.11983/supp-3 Supplemental Information 3 Unsupervised clustering of dominant 5′ capped nucleotides (A) Plot of PC1, PC2, PC3 scores for 17,525 dominant 5′ capped nucleotides clustered using Cross Entropy Clustering (blue = C1 cluster, red = C2 cluster). Plot was generated using the plot3D package in R (Soetaert, 2019) .
(B) Determination of relevant clusters using NbClust (Charrad et al., 2014). Cluster validity was assessed by 27 indices as shown in the bar graph.
Soetaert K. 2019. plot3D: plotting multi-dimensional data. Available at https://CRAN.R-project.org/package=plot3D(accessed on 6 May 2021)

10.7717/peerj.11983/supp-4 Supplemental Information 4 Methylation and acetylation histone marks in the vicinity of dominant 5′ capped nucleotides Score matrices were constructed of normalized occupancies of histone modification marks calculated from chromatin immunoprecipitation sequencing (ChIP-seq) data for 17,525 dominant 5′ capped nucleotides in *Plasmodium falciparum* and 1,000 bp genomic flanks. Plots of average scores for regions in the vicinity of cluster C1 and C2 nucleotides were made using the genomation package (Akalin et al., 2015) in R. To mitigate the effect of extreme values, the top and bottom 5% of scores were clipped using the winsorize function. Data from different stages of the intra-erythrocytic development cycle were plotted on the same axes for each type of histone modification shown. Numbering on the *x*-axes refers to distance (bp) upstream or downstream of 5′ capped nucleotide reference position. Numbering on the y-axes refers to average normalized occupancy. Patterns of histone methylation marks are shown in parts A–D and acetylation marks are shown in parts E–H.
(A) H3K4me1 methylation mark from ChIP-seq data reported in (Tang et al., 2020a).
(B) H3K4me1 methylation mark from ChIP-seq data reported in (Karmodiya et al., 2015).
(C) H3K4me2 methylation mark from ChIP-seq data reported in (Karmodiya et al., 2015).
(D) H3K4me3 methylation mark from ChIP-seq data reported in (Karmodiya et al., 2015).
(E) H3K9 acetylation mark from ChIP-seq data reported in (Karmodiya et al., 2015).
(F) H3K14 acetylation mark from ChIP-seq data reported in (Karmodiya et al., 2015).
(G) H3K18 acetylation mark from ChIP-seq data reported in (Tang et al., 2020a).
(H) H3K27 acetylation mark from ChIP-seq data reported in (Tang et al., 2020a).

10.7717/peerj.11983/supp-5 Supplemental Information 5 Miscellaneous epigenetic marks in the vicinity of dominant 5′ capped nucleotides Score matrices were constructed of normalized occupancies of epigenetic features calculated from epigenetic sequencing data for 17,525 dominant 5′ capped nucleotides in *Plasmodium falciparum* and 1000 bp genomic flanks. Plots of average scores for regions in the vicinity of cluster C1 and C2 nucleotides were made using the genomation package (Akalin et al., 2015) in R. To mitigate the effect of extreme values, the top and bottom 5% of scores were clipped using the winsorize function. Data from different stages of the intra-erythrocytic development cycle were plotted on the same axes for each type of feature shown. Numbering on the *x*-axes refers to the distance (bp) upstream or downstream of 5′ capped nucleotide reference position. Numbering on the *y*-axes refers to the average normalized occupancy.
(A) Nucleosome occupancies from micrococcal nuclease-digested chromatin sequencing data reported in (Kensche et al., 2016). Data are from timepoints hours post-infection (hpi).
(B) H2AZ histone occupancy from chromatin immunoprecipitation sequencing (ChIP-seq) data reported in (Tang et al., 2020a).
(C) H3 histone occupancy from ChIP-seq data reported in (Tang et al., 2020a).
(D) H3 histone occupancy from ChIP-seq data reported in (Karmodiya et al., 2015).
(E) *P. falciparum* GCN5 histone acetyltransferase occupancy from ChIP-seq data reported in (Bhowmick et al., 2020).
(F) RNA polymerase II (RNApolII) occupancy from ChIP-seq data reported in (Karmodiya et al., 2015).
(G) RNApolII occupancy from ChIP-seq data reported in (Lu et al., 2017).

10.7717/peerj.11983/supp-6 Supplemental Information 6 Oligonucleotides Modification codes: 5′ phosphate (/5Phos/), 3′ three-carbon spacer (/3SpC3/). (XXXXXX) refers to Illumina Scriptseq sample index. Sample indices CGATGT, GATCAG, CAGATC, TTAGGC, GCCAAT and CTTGTA were used for library construction in our experiments.

10.7717/peerj.11983/supp-7 Supplemental Information 7 Read alignment summary statistics Paired-end data from transcriptomic libraries sequenced on Illumina HiSeq and MiSeq platforms were pre-processed and aligned to the combined *Plasmodium falciparum* 3D7 v3.2/SIRVomeERCCome (Lexogen) reference genome using HISAT2 (Kim et al., 2019), in the spliced mode guided by the *P. falciparum* / SIRVomeERCCome genome annotation with maximum intron length limited to 5000 bp and other settings as default. Alignment (.bam) files from multiple HiSeq runs of the same library were merged with SAMtools (Li et al., 2009). Summary statistics were generated from alignment files after removal of read2 using SAMtools.

10.7717/peerj.11983/supp-8 Supplemental Information 8 Mismatched base analysis of -1 positions Counts of aligned reads with mismatches to the reference on the first position of the read were made for 12 experimental datasets. Datasets from HiSeq sequencing are shown in Table S3a and MiSeq sequencing in Table S3b. The sum of reads with the same type of mismatched base was made for genomic positions one base upstream of 17,961 dominant 5′ capped nucleotides (”-1” position in reference), and the second group other reference positions. Odds ratio and *P*-values were calculated from Fisher’s exact test. Bonferroni-corrected significant *P*-values are highlighted in yellow.

10.7717/peerj.11983/supp-9 Supplemental Information 9 5′ capped nucleotides in *Plasmodium falciparum* *P*-values of 5′ capped nucleotide enrichment were determined from 12 transcriptomic experiments. Combined *P*-values were determined by Fisher’s method for replicate experiments on the same stage of *P. falciparum* development (ring, trophozoite and schizont; 4 replicates each). Combined *P*-values were transformed to integers and used as input to CAGEr (Haberle et al., 2015) for clustering of nucleotide signals. The outputs from CAGEr are shown as separate tables for ring (S4a), trophozoite (S4b) and schizont (S4c) samples. The outputs were filtered to remove clusters with signals below the significance threshold (tpm ≤ 32). Clusters reported by CAGEr were merged into 17,961 non-overlapping intervals and the dominant_ctss position with the highest count across all three sampled stages was selected as the representative dominant 5′ capped nucleotide for the interval. Merged interval data are shown in S4d. The highest nucleotide count of all CAGEr signals is shown in column 3 (tpm.dominant_ctss). Column 5 (CEC_cluster) is cluster assigned by unsupervised clustering (C1, C2 or not assigned [NA]) using epigenetic data. Column 6 (location) is genomic location with respect to annotated exons (intra or extra-exonic). Columns 7 –9 show mapped read counts at 5′ capped nucleotide position (column 2) from other studies of 5′ capped nucleotides. Counts are from combined data from all experiments in each study: Adjalley (5′ cap sequencing reported by (Adjalley et al., 2016)), Chappell (5UTR-seq reported by (Chappell et al., 2020) and Kensche (SMART enrichment reported by (Kensche et al., 2016).

10.7717/peerj.11983/supp-10 Supplemental Information 10 Full-length 5′ capped transcripts in *Plasmodium falciparum* Transcripts were annotated by FLAIR analysis (Tang et al., 2020b) of direct RNA sequencing data (Lee et al., 2021). Transcripts were classified as 5′ capped if the start position was 20 nt or closer to a dominant 5′ capped nucleotide annotated from full-length enriched cDNA data from one or more studies. Full-length enriched cDNA studies included: CAPture (this study); Adjalley (5′ cap sequencing reported by (Adjalley et al., 2016)), Chappell (5UTR-seq reported by (Chappell et al., 2020) and Kensche (SMART enrichment reported by (Kensche et al., 2016). Correspondence of transcript 5′ end with dominant 5′ capped nucleotide from full-length enriched cDNA data are recorded in Columns 9 –12 (1 = match, 0= no match). FLAIR transcript names are given in Column 3 (”transcript”). For full transcript annotation, see data S1. 5′ capped transcripts were classified according to available gene annotations (1 = match, 0= no match) as coding if they overlapped one or more annotated exons, antisense if they overlapped one or more annotated exons on the opposite strand and novel if they did not overlap any annotated exons.

10.7717/peerj.11983/supp-11 Supplemental Information 11 *Plasmodium falciparum* transcript annotation data Transcripts were annotated from FLAIR analysis (Tang et al., 2020b) of direct RNA sequencing data (Lee et al., 2021).

## Additional Information and Declarations

### DNA Deposition

The following information was supplied regarding the deposition of DNA sequences: Sequencing data generated in this study are available from the National Center for Biotechnology Information (NCBI) Gene Expression Omnibus (GEO) database: GSE103036.

### Data Availability

The following information was supplied regarding data availability: Custom scripts written for analysis of transcriptomic data are available at GitHub: https://github.com/BSI3/5CAPture-seq. Sequencing data generated in this study are available from the National Center for Biotechnology Information (NCBI) Gene Expression Omnibus (GEO) database: GSE103036.
