# Supplementary figures and images for: Identifying transcript 5′ capped ends in Plasmodium falciparum
Source: PeerJ. 2021 Aug 25;9:e11983. doi: 10.7717/peerj.11983 (PMC8401752; doi:10.7717/peerj.11983)

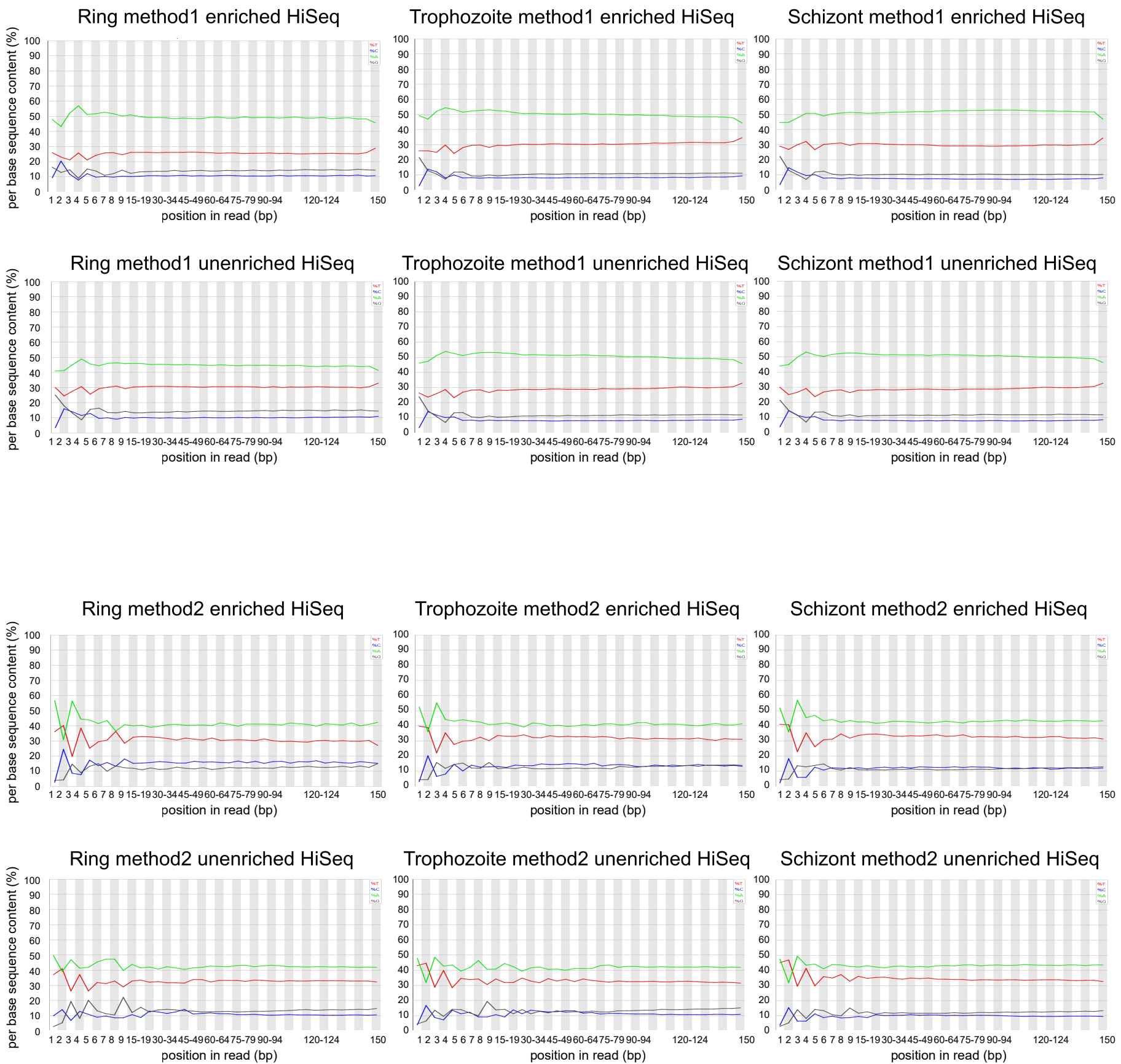

Supplement: Supplemental Information 1 — Plots of average base composition (A, green; T, red; C, blue, G, black) at different positions of read1 were generated using FastQC (Andrews, 2010). The .fastq raw files were preprocessed with Cutadapt 1.18 (Martin, 2011) to remove the homopolymer tail added to cDNA for adapter ligation . Andrews S. 2010. FastQC: a quality control tool for high throughput sequence data. Available at http://www.bioinformatics.babraham.ac.uk/projects/fastqc (accessed on 6 May 2021) [file peerj-09-11983-s001.pdf]

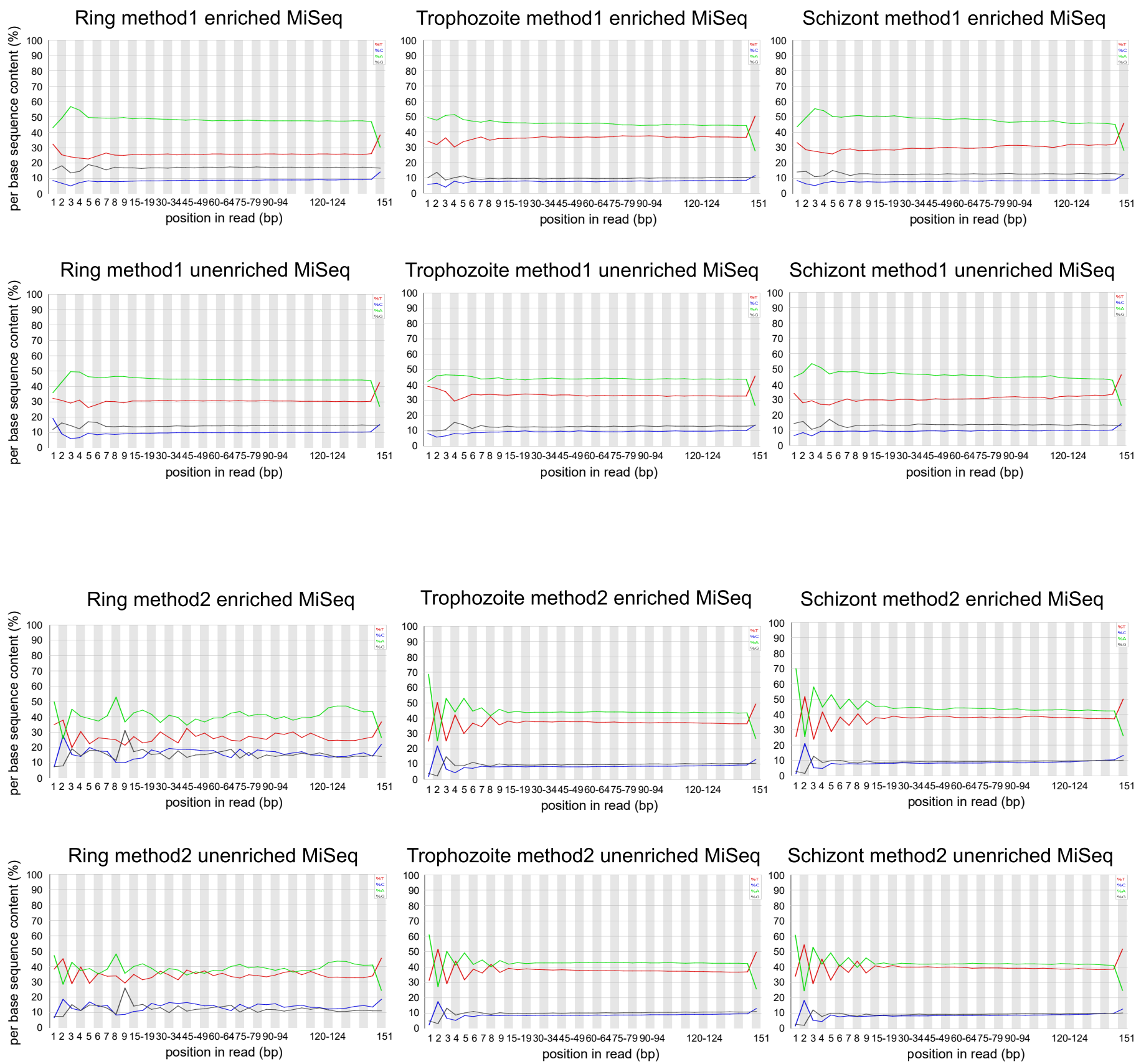

Supplement: Supplemental Information 2 — Plots of average base composition (A, green; T, red; C, blue, G, black) at different positions of read1 were generated from raw .fastq files using FastQC (Andrews, 2010). Andrews S. 2010. FastQC: a quality control tool for high throughput sequence data. Available at http://www.bioinformatics.babraham.ac.uk/projects/fastqc (accessed on 6 May 2021) [file peerj-09-11983-s002.pdf]

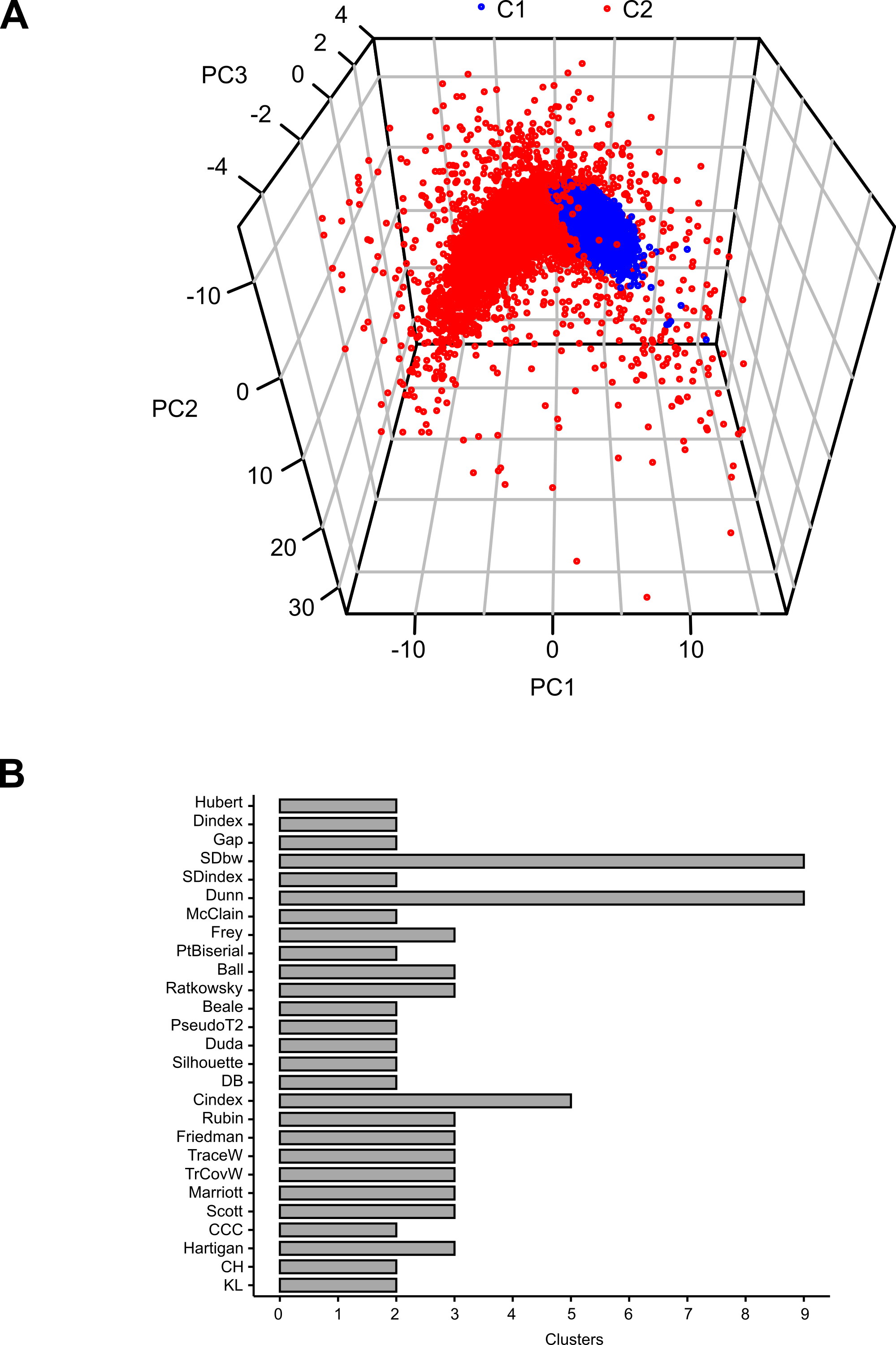

Supplement: Supplemental Information 3 — (A) Plot of PC1, PC2, PC3 scores for 17,525 dominant 5′ capped nucleotides clustered using Cross Entropy Clustering (blue = C1 cluster, red = C2 cluster). Plot was generated using the plot3D package in R (Soetaert, 2019) . (B) Determination of relevant clusters using NbClust (Charrad et al., 2014). Cluster validity was assessed by 27 indices as shown in the bar graph. Soetaert K. 2019. plot3D: plotting multi-dimensional data. Available at https://CRAN.R-project.org/package=plot3D(accessed on 6 May 2021) [file peerj-09-11983-s003.png]

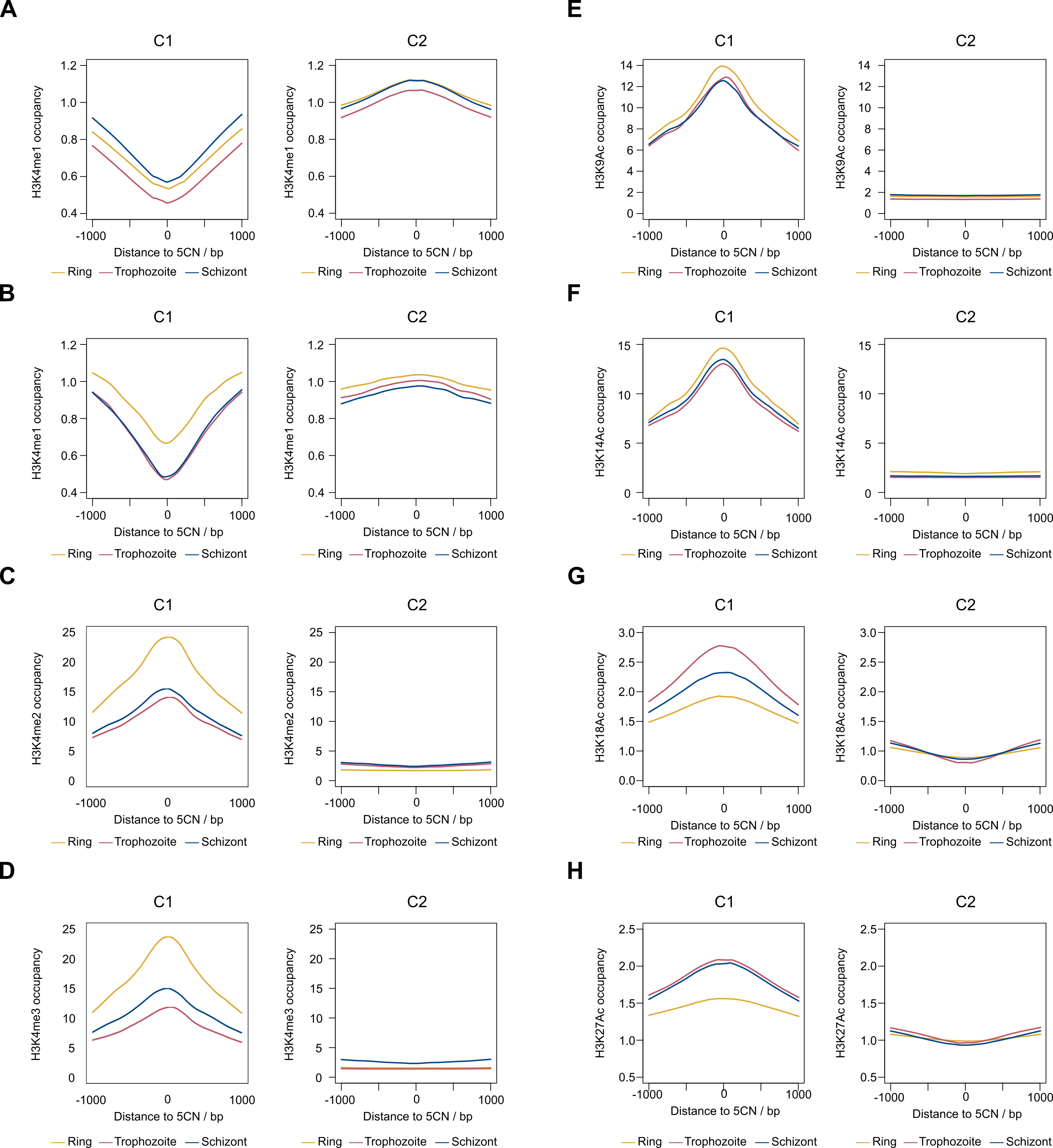

Supplement: Supplemental Information 4 — Score matrices were constructed of normalized occupancies of histone modification marks calculated from chromatin immunoprecipitation sequencing (ChIP-seq) data for 17,525 dominant 5′ capped nucleotides in Plasmodium falciparum and 1,000 bp genomic flanks. Plots of average scores for regions in the vicinity of cluster C1 and C2 nucleotides were made using the genomation package (Akalin et al., 2015) in R. To mitigate the effect of extreme values, the top and bottom 5% of scores were clipped using the winsorize function. Data from different stages of the intra-erythrocytic development cycle were plotted on the same axes for each type of histone modification shown. Numbering on the x-axes refers to distance (bp) upstream or downstream of 5′ capped nucleotide reference position. Numbering on the y-axes refers to average normalized occupancy. Patterns of histone methylation marks are shown in parts A–D and acetylation marks are shown in parts E–H. (A) H3K4me1 methylation mark from ChIP-seq data reported in (Tang et al., 2020a). (B) H3K4me1 methylation mark from ChIP-seq data reported in (Karmodiya et al., 2015). (C) H3K4me2 methylation mark from ChIP-seq data reported in (Karmodiya et al., 2015). (D) H3K4me3 methylation mark from ChIP-seq data reported in (Karmodiya et al., 2015). (E) H3K9 acetylation mark from ChIP-seq data reported in (Karmodiya et al., 2015). (F) H3K14 acetylation mark from ChIP-seq data reported in (Karmodiya et al., 2015). (G) H3K18 acetylation mark from ChIP-seq data reported in (Tang et al., 2020a). (H) H3K27 acetylation mark from ChIP-seq data reported in (Tang et al., 2020a). [file peerj-09-11983-s004.png]

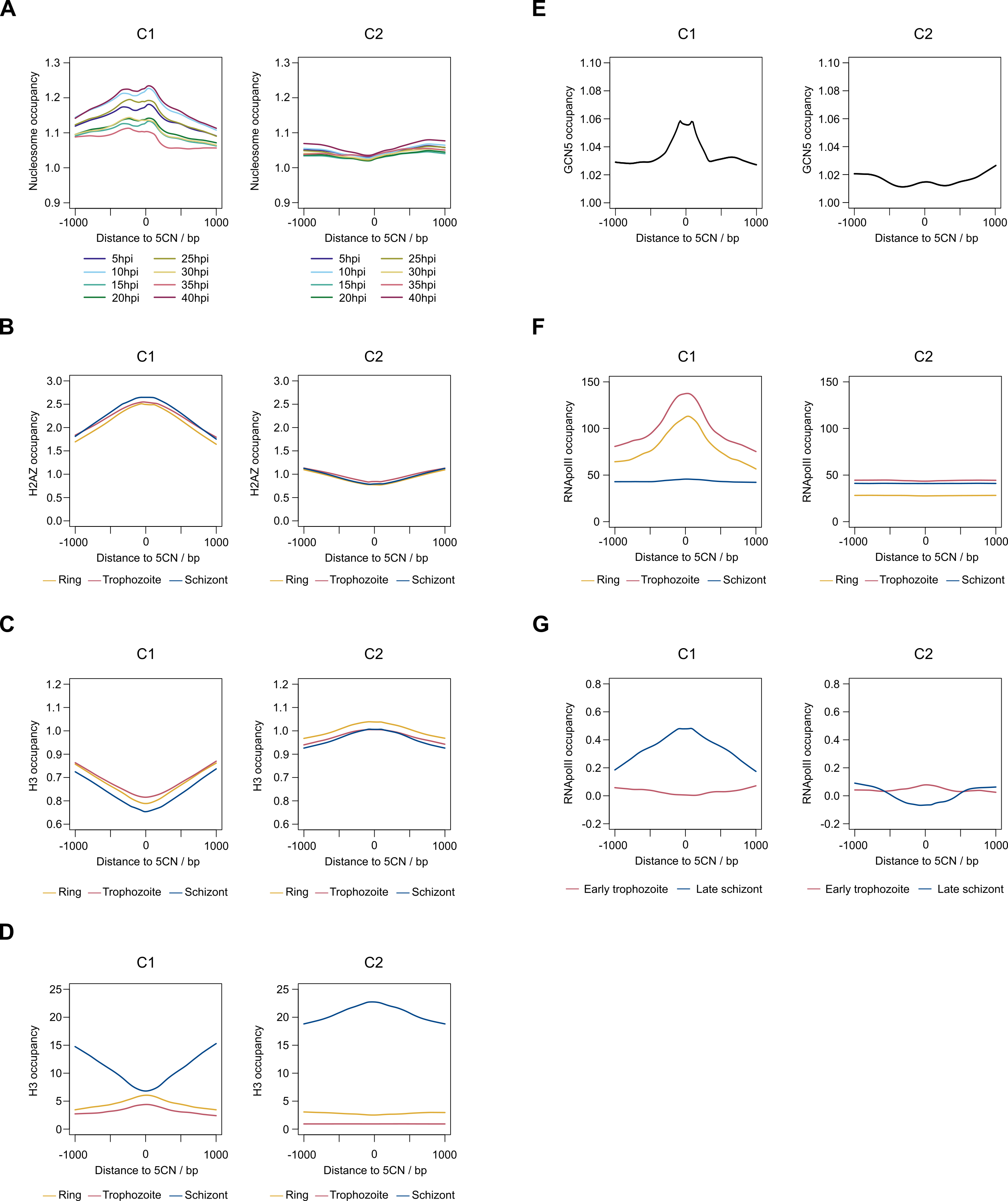

Supplement: Supplemental Information 5 — Score matrices were constructed of normalized occupancies of epigenetic features calculated from epigenetic sequencing data for 17,525 dominant 5′ capped nucleotides in Plasmodium falciparum and 1000 bp genomic flanks. Plots of average scores for regions in the vicinity of cluster C1 and C2 nucleotides were made using the genomation package (Akalin et al., 2015) in R. To mitigate the effect of extreme values, the top and bottom 5% of scores were clipped using the winsorize function. Data from different stages of the intra-erythrocytic development cycle were plotted on the same axes for each type of feature shown. Numbering on the x-axes refers to the distance (bp) upstream or downstream of 5′ capped nucleotide reference position. Numbering on the y-axes refers to the average normalized occupancy. (A) Nucleosome occupancies from micrococcal nuclease-digested chromatin sequencing data reported in (Kensche et al., 2016). Data are from timepoints hours post-infection (hpi). (B) H2AZ histone occupancy from chromatin immunoprecipitation sequencing (ChIP-seq) data reported in (Tang et al., 2020a). (C) H3 histone occupancy from ChIP-seq data reported in (Tang et al., 2020a). (D) H3 histone occupancy from ChIP-seq data reported in (Karmodiya et al., 2015). (E) P. falciparum GCN5 histone acetyltransferase occupancy from ChIP-seq data reported in (Bhowmick et al., 2020). (F) RNA polymerase II (RNApolII) occupancy from ChIP-seq data reported in (Karmodiya et al., 2015). (G) RNApolII occupancy from ChIP-seq data reported in (Lu et al., 2017). [file peerj-09-11983-s005.png]
